# Flavonoids regulate LDLR through different mechanisms tied to their specific structures

**DOI:** 10.1016/j.jlr.2024.100539

**Published:** 2024-03-29

**Authors:** Katrine Bjune, Pia Skovholt Halvorsen, Helle Wangensteen, Trond P. Leren, Martin Prøven Bogsrud, Thea Bismo Strøm

**Affiliations:** 1 Unit for Cardiac and Cardiovascular Genetics, Department of Medical Genetics, Oslo University Hospital, Oslo, Norway; 2 Section for Pharmaceutical Chemistry, Department of Pharmacy, University of Oslo, Oslo, Norway

**Keywords:** polyphenols, antioxidants, structure, lipoproteins, cardiovascular disease, nutrition, metabolism, atherosclerosis

## Abstract

Flavonoids, polyphenolic compounds found in plant-based diets, are associated with reduced risk of cardiovascular disease and longevity. These components are reported to reduce plasma levels of low-density lipoprotein (LDL) through an upregulation of the LDL receptor (LDLR), but the mechanism is still largely unknown. In this study, we have systematically screened the effect of 12 flavonoids from six different flavonoid subclasses on the effect on LDLR. This paper provides an in-depth analysis on how these flavonoids affect LDLR regulation and functionality. We found that most but not all of the tested flavonoids increased *LDLR* mRNA levels. Surprisingly, this increase was attributed to different regulatory mechanisms, such as enhanced *LDLR* promoter activity, *LDLR* mRNA stabilization, or LDLR protein stabilization, of which specific effectual parts of the flavonoid molecular structure could be assigned. These types of comparative analysis of various flavonoids enhance clarity and deepen the understanding of how the different structures of flavonoids affect LDLR regulation. Our data offer useful insights that may guide future research in developing therapeutic approaches for cardiovascular health.

Low-density lipoprotein (LDL) is the primary cholesterol transporting lipoprotein particle, carrying approximately 70% of circulating plasma cholesterol (1). The level of plasma LDL-cholesterol is predominantly regulated by the LDL receptor (LDLR), which facilitates the clearance of LDL particles through receptor-mediated endocytosis (2). Consequently, genetic variants resulting in decreased number or decreased functionality of the LDLR lead to elevated levels of LDL-cholesterol in plasma, as demonstrated in patients with familial hypercholesterolemia (FH) (OMIM#14390) (3, 4). The life-long elevated levels of LDL cholesterol caused by variants in *LDLR* in FH patients culminates in premature cardiovascular events and death (5, 6, 7). Various therapies aimed at increasing the amount of LDLRs, such as statins and antibodies against proprotein convertase subtilisin/kexin type 9 (PCSK9), are effective in reducing cardiovascular disease incidence (8, 9). However, there is still an unmet need to identify new therapeutic strategies for treating hypercholesterolemia, as specific patients groups, like the FH patients, often fail to reach their cholesterol level targets or may suffer from side effects from the currently available lipid-lowering drugs.

Regulation of *LDLR* expression is a multifaceted process crucial for normal cellular function. Transcription of *LDLR* is primarily controlled by sterol regulatory element-binding protein-2 (SREBP2), a transcription factor initially formed as an inactive precursor bound to the endoplasmic reticulum membrane (10). Upon low intracellular cholesterol levels, SREBP2 is transported by SREBP cleavage-activating protein to the Golgi apparatus, where it undergoes sequential proteolytic cleavages, releasing the active NH_2_-terminal domain (11). This active domain of SREBP-2 then enters the nucleus, binds to the sterol regulatory element (*SRE*) in the *LDLR* promoter, and induces *LDLR* expression. In addition to regulation on the transcriptional level, *LDLR* mRNA levels are also influenced by factors affecting its stability. The *LDLR* mRNA has a relatively short half-life of approximately two hours, and the 3' untranslated region’ contains binding sites for proteins affecting the stabilization of the mRNA, thus increasing or decreasing the mRNA half-life (12, 13). Furthermore, LDLR protein levels are also affected by posttranslational regulation either through interaction with PCSK9 or inducible degrader of LDLR, both of which lead to subsequent degradation of the LDLR protein (14, 15).

Recent research indicates that specific flavonoids may alter *LDLR* expression and LDLR function, presenting potential therapeutic opportunities for preventing cardiovascular disease (16). Flavonoids are a group of polyphenolic compounds produced in virtually all land-based green plants (17). They are abundant in certain vegetables, fruits, juices, tea, wine, and grains and are acknowledged for their potential health advantages (18, 19). Flavonoids are highly oxygenated with OH-groups, OCH_3_ groups, or glycosyl groups at specific carbon atoms on the A-, B-, and C-rings, which gives rise to a high number of structures with different biochemical properties (18, 19), and are classified into subclasses depending on the level of oxidation of the C-ring (20). The flavonoids exhibit a broad spectrum of biological activities and are considered to play a role in lipid metabolism, particularly improving cardiovascular health and longevity. Although some flavonoids like hesperetin, naringenin, kaempferol, quercetin, nobiletin, epigallocatechin gallate, and genistein have been associated with LDLR modulation in earlier studies (21, 22, 23, 24, 25, 26, 27), a comprehensive understanding of the flavonoid–LDLR interactions, structure and function relationships, and their impact on the amount of LDLR remains elusive.

In this study, we extend the current knowledge of the effects of flavonoids on the LDLR by examining a comprehensive set of flavonoids from the different subclasses, also including compounds which, to our knowledge, have not been previously studied in relation to LDLR regulation. Using cell-based assays, we have meticulously investigated not only the potential effects of these flavonoids on LDLR protein levels but also delve into the regulatory mechanisms, including effects on promoter activity and mRNA and protein stabilization. This research aims to elucidate the complex interactions between flavonoids and LDLR, thereby establishing a robust foundation for future studies and possibly new therapeutic strategies.

## Materials and Methods

### Flavonoids studied

**Fig. 1:**
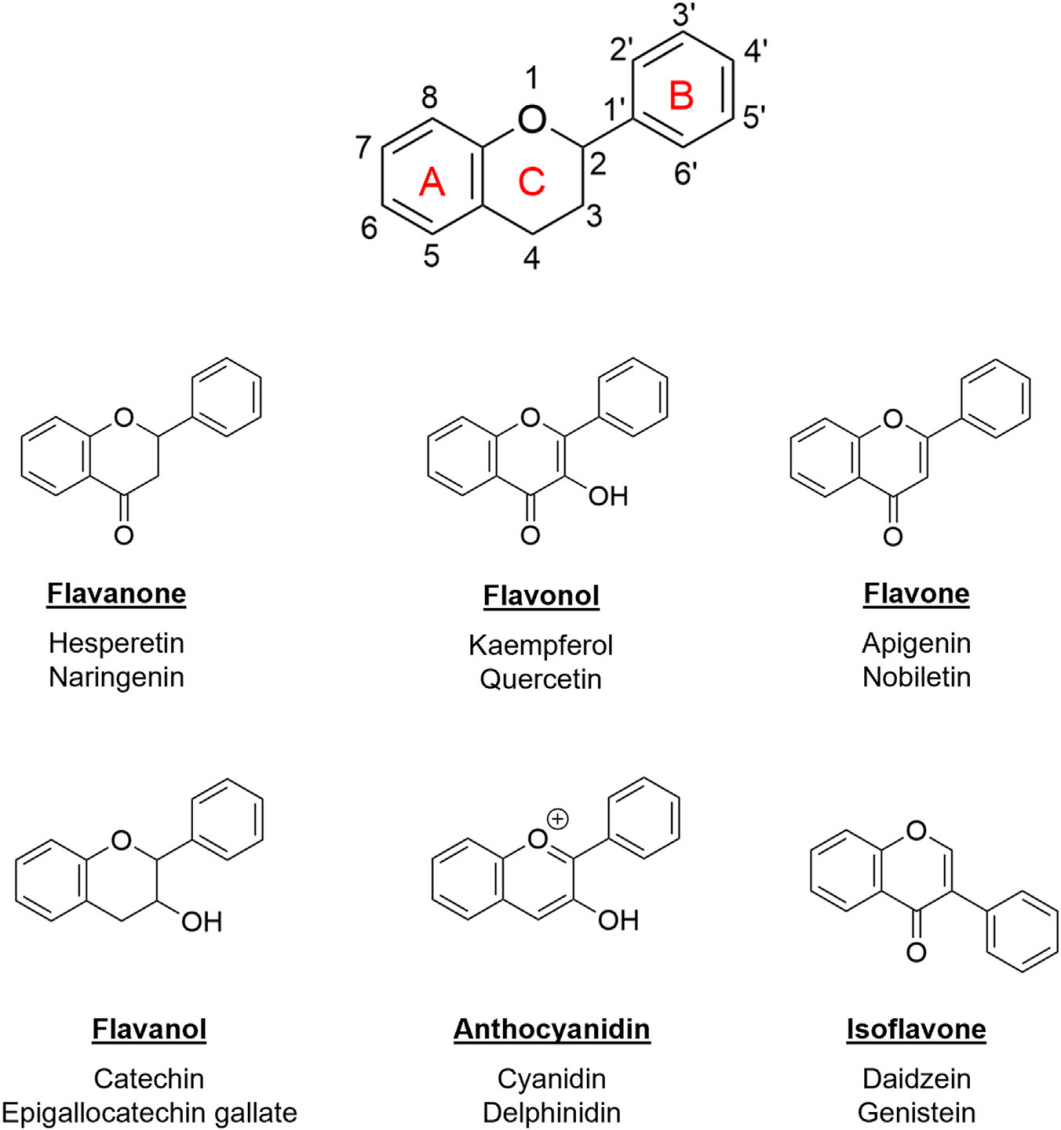
Flavonoid structure. The basic skeleton structure of flavonoids with ring numbering, the core structures of the six major flavonoid subclasses, and the twelve flavonoids included in this study.

Flavonoids from six subclasses were included in this study: flavanones, flavonols, flavones, flavanols, anthocyanidins, and isoflavones (Fig. 1). Within the flavanone and flavonol groups, hesperetin and naringenin, as well as quercetin and kaempferol, have been identified to positively influence LDLR levels (22, 24, 25, 26). Beside these, three other flavonoids known to enhance LDLR levels are the flavone nobiletin, the flavanol epigallocatechin gallate, and the isoflavone genistein (21, 23, 27). To our knowledge, no pure forms of flavonoids in the anthocyanidin subclass have been shown to impact the LDLR. To delve deeper into the effect of flavonoids on the LDLR, we have also included five other flavonoids: apigenin (flavone), catechin (flavanol), cyanidin (anthocyanidin), delphinidin (anthocyanidin), and daidzein (isoflavone). These flavonoids have no previously documented effect on LDLR protein levels, and by incorporating these, we have two components from each of the six subclasses of flavonoids.

### Reagents and antibodies

Epigallocatechin gallate (E4143), daidzein (D7802), delphinidin chloride (43725), catechin (43412), apigenin (10798), kaempferol (60010), genistein (G6649), quercetin (Q4951), and hesperetin (W431300) were obtained from Sigma Aldrich (St. Louis, MO). Naringenin (S2394) and nobiletin (S2333) were obtained from Selleck Chemicals (Houston, TX), and cyanidin chloride (sc-202559) was from BioMed (Heidelberg, Germany). Most flavonoids had a purity above 98%. However, epigallocatechin gallate, delphinidin, apigenin, hesperetin, and quercetin had a purity above 95%. The flavonoids were dissolved in dimethyl sulfoxide (DMSO; Sigma Aldrich), contained under argon gas and added to cells in a low light environment. Actinomycin D (A1410), cycloheximide, and dithiothreitol (D0632) were obtained from Sigma Aldrich. Antibody against LDLR (3839-100) was purchased from BioVision (Milpitas, CA), and antibody against β-actin (AB8227) was from Abcam (Cambridge, UK). Anti-LDLR IgG-C7 (#61087) used in flow cytometry was purchased from Progen Biotechnik GmbH (Heidelberg, Germany), while antibody against Poly (ADP-ribose) polymerase (PARP) was obtained from Cell signaling (Danvers, MA), and staurosporine was from Sigma-Aldrich (S4400). 3-[4,5-dimethylthiazol-2-yl]-2,5 diphenyl tetrazolium bromide (MTT) assay kit was purchased from Abcam (ab21091), and Triton X-100 was from Sigma-Aldrich.

### Cell cultures

HepG2 cells (European Collection of Cell Cultures, Salisbury, UK), were cultured on collagen-coated culture vessels (BD Biosciences, San Jose, CA) in HyClone Minimum Essential Medium (GE Healthcare Life Sciences, Pittsburg, PA) containing 10% fetal bovine serum (Sigma-Aldrich), 2 mM L-glutamine (Sigma-Aldrich), 50 U/ml penicillin (GE Healthcare Life Sciences), 50 *μ*g/ml streptomycin (GE Healthcare Life Sciences), and nonessential amino acids (Biowest, Nuaillé, France). The cells were grown in monolayer in an atmosphere of 5% CO_2_ at 37°C. All compounds were added to the cells with a DMSO concentration of 0.1% (v/v). To evade possible DMSO effects, control cells were treated with DMSO (vehicle) alone at final concentrations of 0.1%.

### Western blot analysis

Cells were lysed in Triton X-100 lysis buffer (20 mM Tris [pH 7.5], 100 mM NaCl, 1% Triton X-100, 10 mM EDTA, and Complete Protease Inhibitor Cocktail [Roche, Basel, Switzerland]). Equal amounts of proteins were separated by 4–20% sodium dodecyl sulfate polyacrylamide gel electrophoresis. After transfer to a polyvinylidene difluoride membrane (Bio-Rad, Hercules, CA), proteins were detected by the use of standard immunoblotting procedures. The band intensities were quantified by the use of Chemidoc Touch Imaging System (Bio-Rad).

### Quantitative real-time PCR

Total RNA was extracted from cells using Monarch total RNA miniprep kit (T2010S; New England Biolabs, Ipswich, MA). cDNA was synthesized with the AffinityScript QPCR cDNA Synthesis Kit (Agilent Technologies, Santa Clara, CA). Quantitative real-time PCR (qPCR) was performed using Luna universal master mix (M30043; New England Biolabs) on Mx3005P QPCR system (Agilent Technologies). The PrimeTime Predesigned qPCR Assay probes used (Integrated DNA Technologies, Coralville, IA) and were LDLR: hs.pt.53.24904291, transferrin receptor protein: Hs.PT.58.22906586, 3-hydroxy-3-methylglutaryl-coenzyme A reductase (HMGCR): hs.pt.56.41105492, PCSK9: hs.pt.53a.19328431, fatty acid synthase: Hs.PT.58.20384174, SREBP-1c: Hs.PT.58.25035932, SREBP-2: Hs.PT.58.4533543, and ceraldehyde-3-phosphate dehydrogenase: Hs.PT.39a.22214836. 25 ng DNA template was used, and the samples were analyzed as duplicates in four separate experiments. The housekeeping gene *glyceraldehyde-3-phosphate dehydrogenase* was used for normalizing the amount of target mRNA. Relative mRNA expression was calculated using the 2^−ΔΔ*Ct*^ method (28).

### Plasmids, transfection, and reporter assay

The luciferase reporter plasmid containing the *LDLR* promoter sequence −1563 to +58, pLR1563-luc (29), was a gift from Dr Youngmi Kim Pak (University of Ulsan College of Medicine, Seoul, Republic of Korea). The LDLR promoter containing a mutated *SRE* motif have GG instead of CC in the sequence ATCAACCCCAC. For plasmid transfections, cultured HepG2 cells were transfected using FuGENE HD (Promega, Madison, WI) according to the manufacturer’s instructions. A ratio between FuGENE HD and plasmid DNA of 4.5:1 was used. Cells transfected with empty vector were used as a control. Flavonoids were added to the cells 24 h after transfection and harvested after 6 h or 18 h. Analysis of reporter gene activities was performed by the use of Dual-Luciferase Reporter Assay (E1910; Promega), according to the manufacturer’s instructions. For measurement of *LDLR* promoter activity, cells were cotransfected with pLR1563-luc and the Renilla luciferase plasmid, phRL (Promega) at a ratio of 9:1.

### MTT assay

The level of cell growth and proliferation was determined by using the MTT assay kit (ab211091; Abcam). The MTT assay is a colorimetric assay where viable cells reduce the yellow tetrazolium dye MTT to insoluble formazan crystals, which are purple. This assay was utilized to evaluate the cytotoxicity of different flavonoids on HepG2 cells. The assay was performed according to the manufacturer’s instructions with some modifications. Briefly, HepG2 cells were seeded in a 48-well plate at 30% confluency instead of in a 96-well plate. Cells were treated with either vehicle or various concentrations of different flavonoids overnight at 37°C with 5% CO_2_. The following day, serum-containing medium was replaced with serum-free medium, and MTT reagent was added to the cell cultures, followed by incubation at 37°C for 3 h. After incubation, MTT solvent was added, and the cultures were incubated for an additional 15 min. Cell viability was determined by measuring absorbance at absorbance 590 nm.

### Analysis of cell-surface LDLR and LDL internalization by flow cytometry

For detection of the amount of cell-surface LDLR, HepG2 cells were harvested using Non-Enzymatic Cell Dissociation Solution (Sigma-Aldrich). The samples were washed twice with staining buffer (phosphate-buffered saline + 0.5% bovine serum albumin) and incubated with mouse monoclonal anti-LDLR antibody IgG-C7 (1:20 dilution in Staining Buffer) at 4°C for 1 h. Cells were then washed three times and incubated with Alexa Fluor 488-conjugated goat anti-mouse IgG antibody (1:400 dilution in Staining Buffer; #A11001; Invitrogen Carlsbad, CA) at 4°C for 30 min in the dark. After antibody incubation, cells were washed twice with Staining Buffer, resuspended in phosphate-buffered saline, and analyzed on a BD Accuri™ C6 Plus flow cytometer (BD Biosciences) for quantification of Alexa Fluor 488 fluorescence. LDL (density 1.019–1.063 g/ml) was isolated by ultracentrifugation of human serum and labeled with fluorescent 1,1′-dioctadecyl-3,3,3′,3′-tetramethylindodicarbocyanine perchlorate (DiD; Invitrogen) as described by Pitas *et al.* (30). To measure LDLR internalization activity, cells were incubated with DiD-LDL (10 μg/ml) at 37°C for 2 h, washed twice in staining buffer, and harvested by trypsinization. The amount of internalized DiD-LDL was measured at 647 nm by flow cytometry.

### Optimizing flavonoid concentration

Based on previous publications (21, 22, 23, 24, 25, 26, 27), the concentration of each flavonoid was optimized by assessing LDLR protein level, level of induced apoptosis (supplemental Figs. S1 and S2), and cell viability (supplemental Fig. S3) following varying overnight dose treatments. Concentrations ranged from 1 to 200 μM. Two to three flavonoid concentrations were selected based on their impact on cell vitality, the amount of LDLR, and apoptosis after 6-h treatments (supplemental Figs. S4 and S5). Additionally, LDLR surface levels and LDL uptake was assessed to determine the functional effects of the flavonoids on LDLR (supplemental Fig. S6). The optimal concentrations, which prioritized both cell wellbeing and effects on the amount of LDLRs, LDLR-mediated uptake of LDL, and LDLR surface levels, were chosen. The optimal concentrations of each flavonoids were as follows: hesperetin 150 μM, naringenin 150 μM, kaempferol 50 μM, quercetin 75 μM, apigenin 10 μM, nobiletin 5 μM, catechin 50 μM, epigallocatechin gallate 100 μM, cyanidin 50 μM, delphinidin 100 μM, daidzein 50 μM, and genistein 50 μM.

### Statistical analyses

**Fig. 2:**
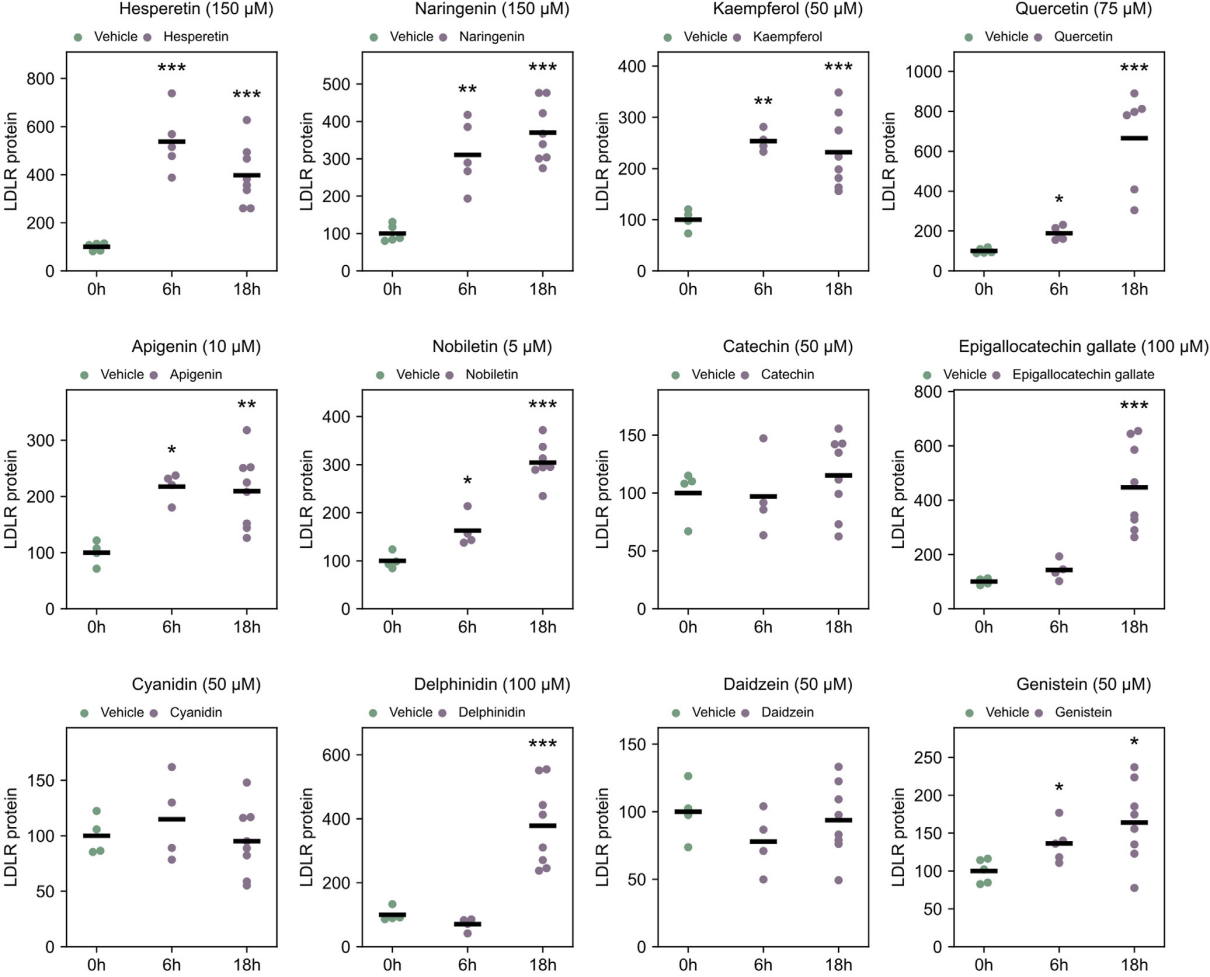
LDLR protein levels after 6 h and 18 h treatment with different flavonoids. HepG2 cells were treated with vehicle or different flavonoids for 6 h or overnight (18 h) before harvesting and determination of LDLR and β-actin levels by Western blot analyses. LDLR protein levels were corrected for by β-actin levels and plotted relative to vehicle-treated control which were assigned a value of 100. Vehicle-treated controls (DMSO) (0 h) and different flavonoids (6 and 18 h) are presented in a dot plot where each dot represents an outcome of an experiment, and a black line represents the mean value (∗*P* < 0.05, ∗∗*P* < 0.01, ∗∗∗*P* < 0.001, two-tailed *t* test vs. vehicle-treated cells). The concentrations used were based on earlier publications and data found in supplemental data.

**Fig. 3:**
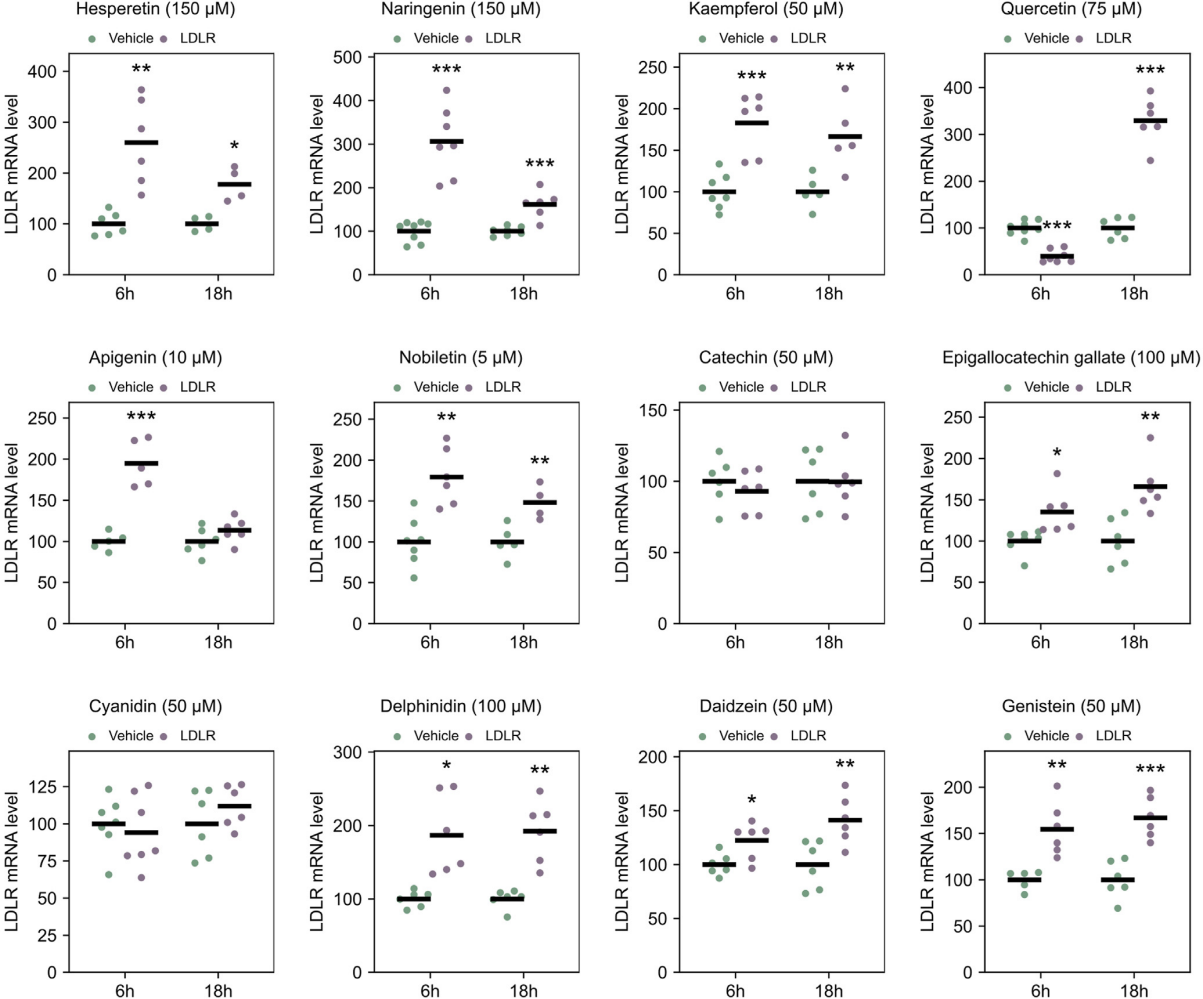
*LDLR* mRNA levels after 6 h or 18 h treatment with different flavonoids. HepG2 cells were treated with vehicle or different flavonoids for 6 h or overnight (18 h) before harvesting and determination of *LDLR* mRNA levels by qPCR. *LDLR* mRNA levels were plotted relative to that of the vehicle treated control cells. Vehicle treated controls (DMSO) (light blue) were assigned a value of 100 and together with the different flavonoids (dark blue) are presented in a dot plot where each dot represents an outcome of an experiment, and a black line represents the mean value (∗*P* < 0.05, ∗∗*P* < 0.01, ∗∗∗*P* < 0.001, two-tailed *t* test vs. vehicle treated cells).

**Fig. 4:**
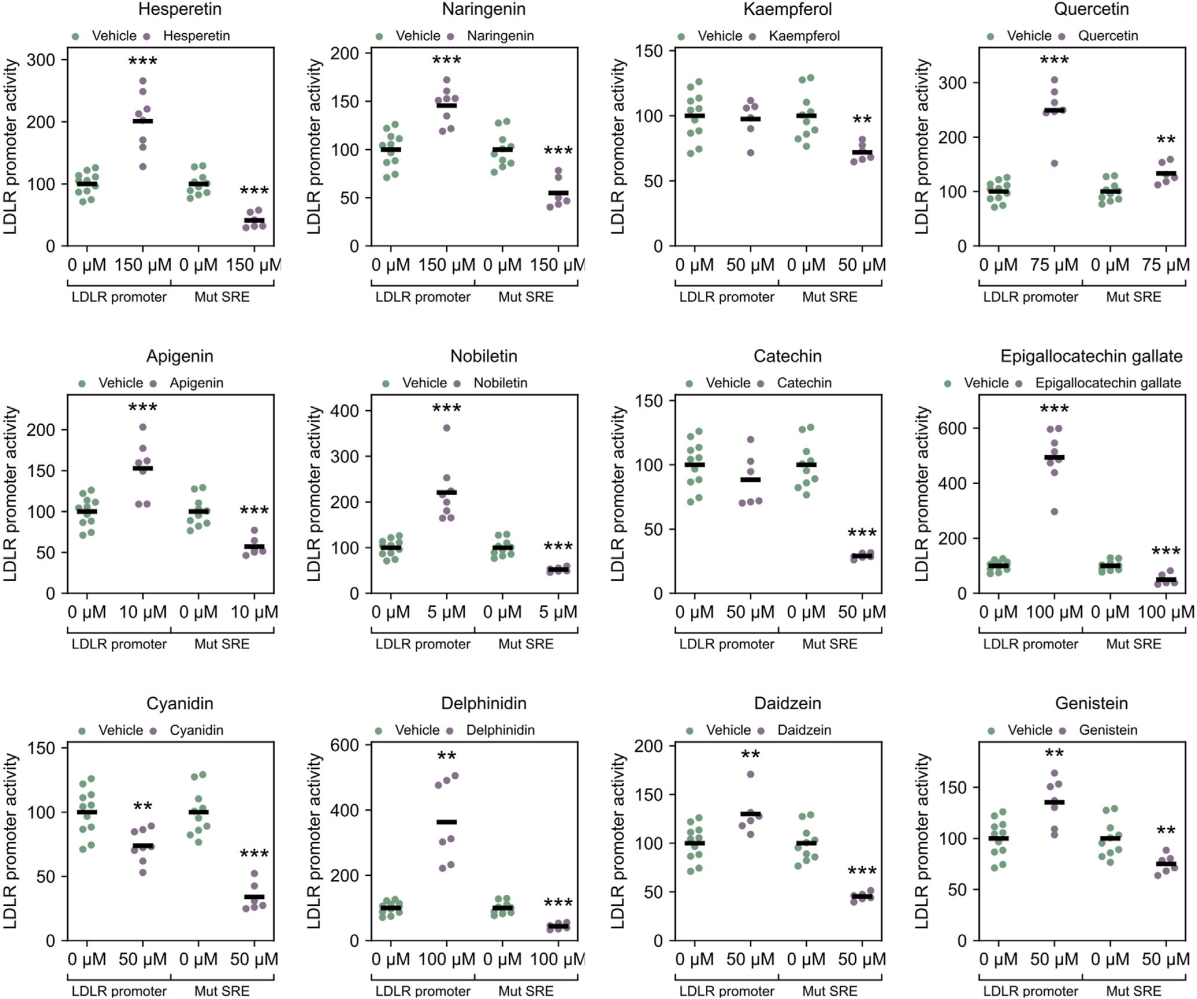
*LDLR* promoter activity after treatment with different flavonoids. HepG2 cells were transfected with the pLR1563-luc plasmid or the same plasmid with a mutated *SRE* sequence and the Renilla luciferase plasmid. Twenty-four hours after transfection, the cells were washed and treated with vehicle or different flavonoids overnight (18 h). The cells were then harvested for analysis of luciferase activity. The luciferase activity was corrected by Renilla luciferase activity and plotted relative to the vehicle-treated control cells. Vehicle treated controls (DMSO) (0 μM) and different flavonoids are presented in a dot plot where each dot represents an outcome of an experiment, and a black line represents the mean value (∗∗*P* < 0.01, ∗∗∗*P* < 0.001, two-tailed *t* test vs. vehicle treated cells).

**Fig. 5:**
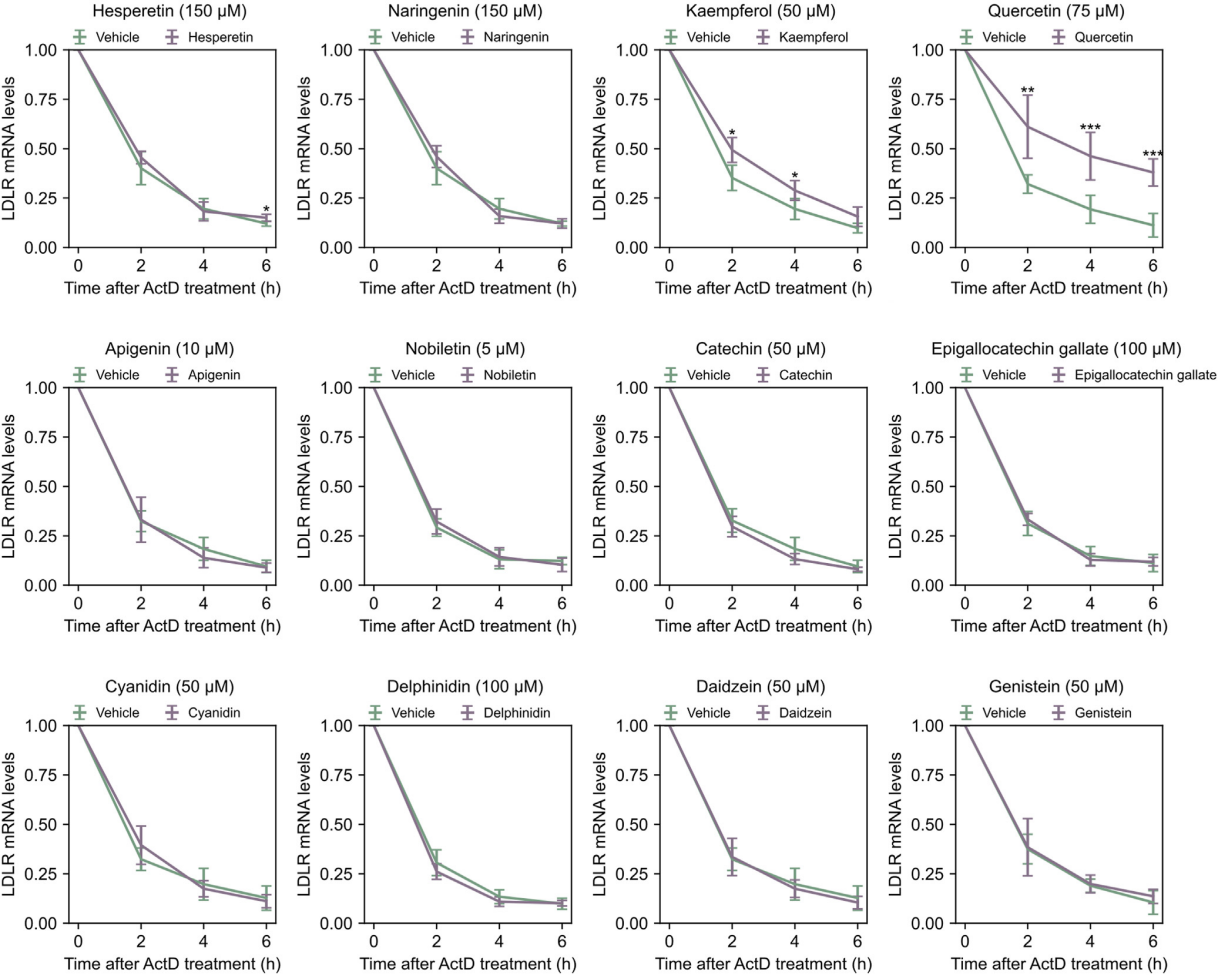
*LDLR* mRNA stability after treatment with different flavonoids. HepG2 cells were treated with vehicle or different flavonoids overnight (18 h). 5 ug/ml Actinomycin D (Act D) was added to the cultures and harvested after 0, 2, 4, and 6 h. After harvesting, the samples were analyzed by qPCR for *LDLR* and *GAPDH* mRNA levels. *LDLR* mRNA levels were corrected for by GAPDH levels and plotted relative to the internal 0-h control. Vehicle-treated controls (DMSO) (light blue) and different flavonoids (dark blue) are presented as mean +SD from six independent experiments (individual data points are found in supplemental Fig. S8) (∗*P* < 0.05, ∗∗*P* < 0.01, ∗∗∗*P* < 0.001, two-tailed *t* test vs. vehicle treated cells).

**Fig. 6:**
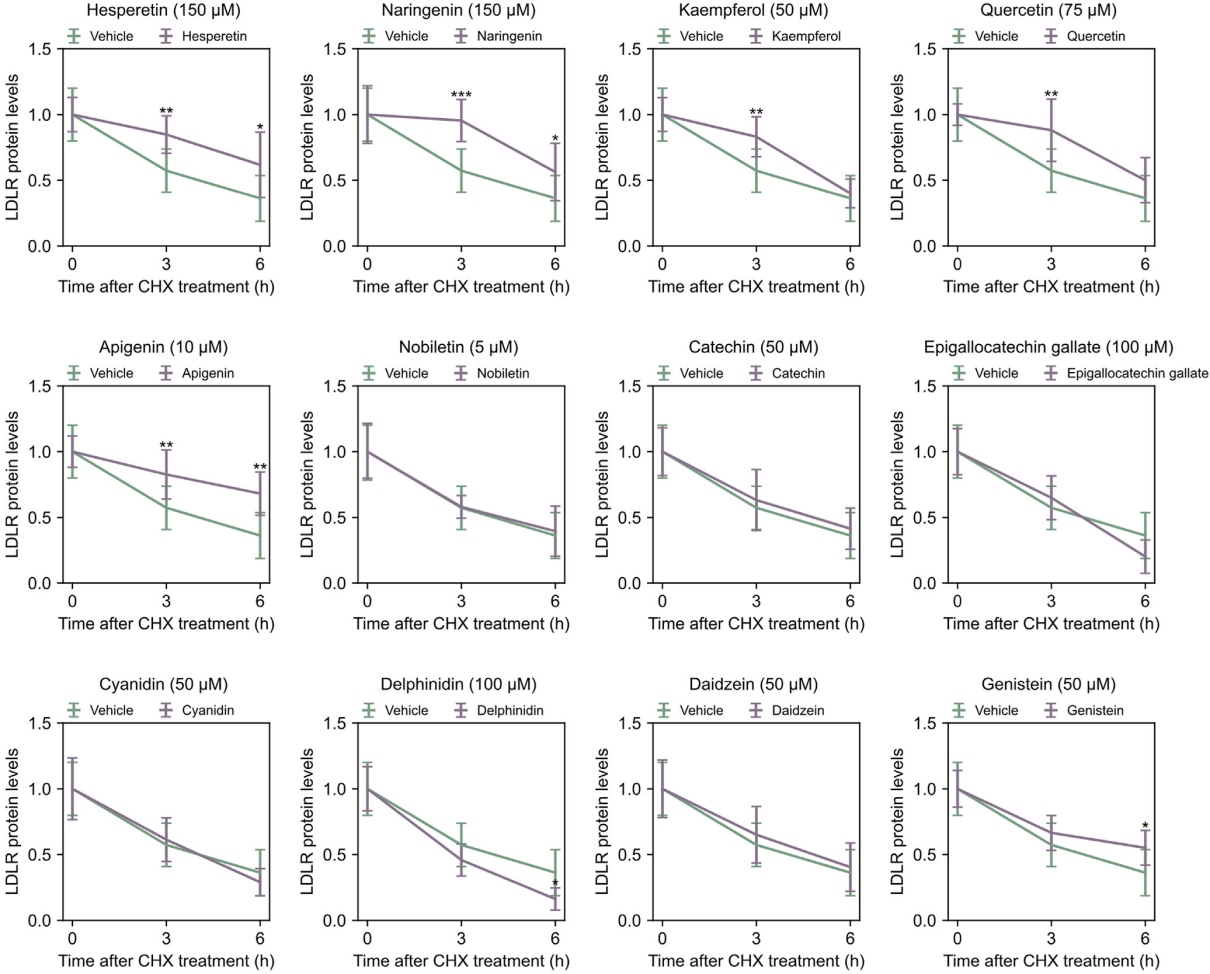
LDLR protein stability after treatment with different flavonoids. HepG2 cells were treated with vehicle or different flavonoids for 4 h. 50 ug/ml cycloheximide (CHX) was added to the cultures and harvested after 0, 3, and 6 h. After harvesting, the samples were analyzed by Western blot for LDLR and β-actin levels (one representative plot for each flavonoid and vehicles are included in supplemental Fig. S9). LDLR protein levels were corrected for by β-actin levels and plotted relative to the internal 0-h control. Vehicle-treated controls (DMSO) (light blue) and different flavonoids (dark blue) are presented as mean +SD from six independent experiments (individual data points are found in supplemental Fig. S10) (∗*P* < 0.05, ∗∗*P* < 0.01, ∗∗∗*P* < 0.001, two-tailed *t* test vs. vehicle treated cells).

All data in Fig. 2, Fig. 3, Fig. 4 are displayed as dot plots containing a black line marking the mean value, while data in Figs. 5 and 6 are displayed as mean ± SD. To evaluate the effects of flavonoid treatment on cell populations, our statistical analysis was tailored to the specific experimental design. For experiments assessing a single concentration of flavonoids against vehicle-treated controls (Figs. 3 and 4, and supplemental Fig. S7), we initially conducted an F-test to assess the equality of variances. Depending on this assessment, data with equal variances were analyzed using Student's *t* test, while Welch's *t* test was employed for data with unequal variances. When multiple flavonoid concentrations were tested (Figs. 5 and 6, supplemental Figs. S1, S3, S4, and S6), we applied ANOVA with Tukey's post hoc test to ascertain significant differences between groups. These statistical analyses provided a robust framework for interpreting our data, ensuring that our findings were both comprehensive and reliable. A *P*-value < 0.05 was considered statistically significant (∗) (∗∗*P* < 0.01 and ∗∗∗*P* < 0.001). The LDLR mRNA half-life was calculated using least squares regression.

### Illustrations and figures

**Fig. 7:**
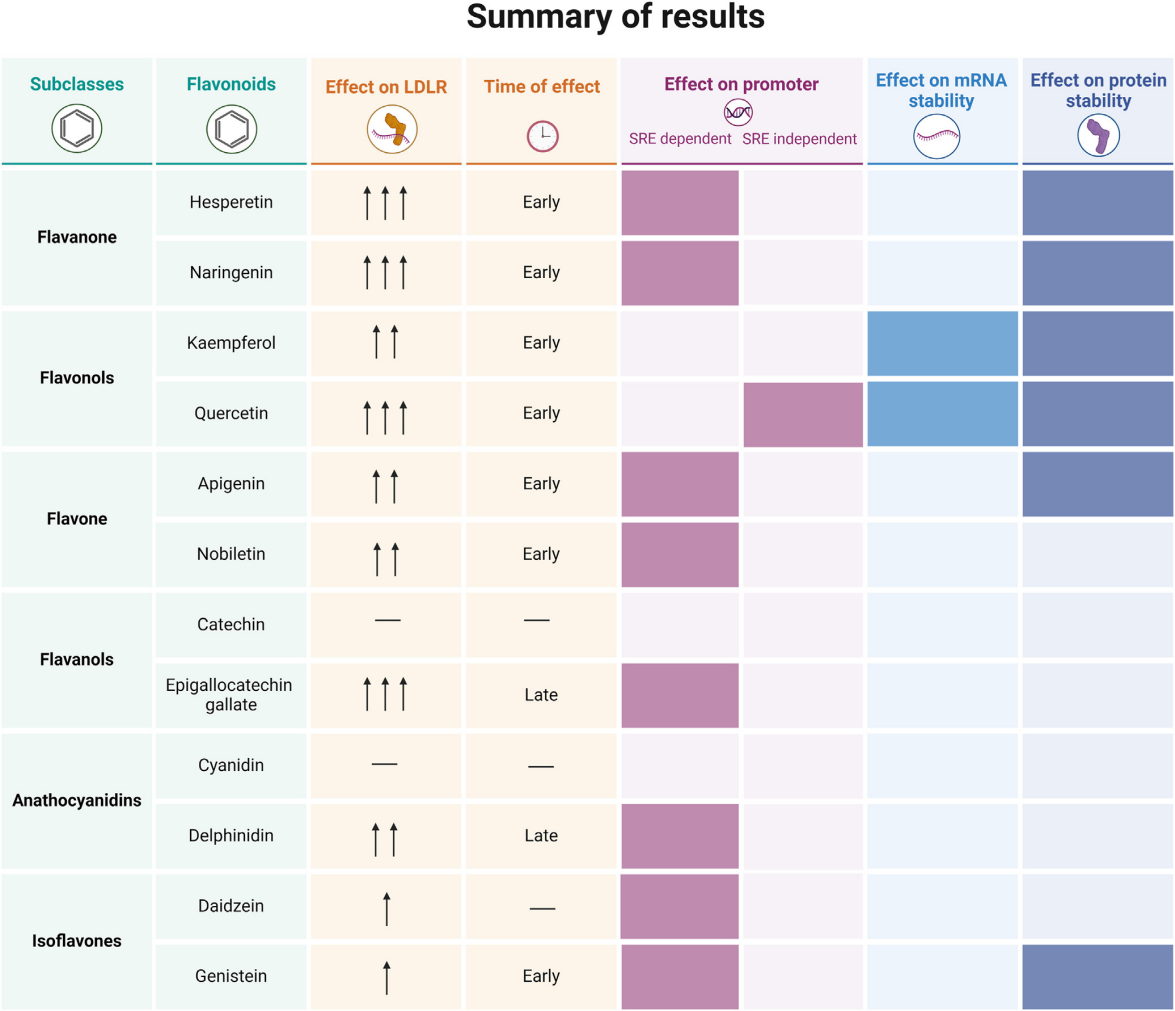
A summary of results. The table lists the flavonoid subclasses, names, effects on the amount of LDLR, time of effect, and their LDLR-regulating mechanisms. In the “effect on LDLR” column, a dash indicates no observed effect, while one to three upward-pointing arrows denote the intensity of the effect. The criteria for assigned arrows were as follows: *1)* show a significant increase in *LDLR* mRNA levels, *2)* a rise in LDLR protein levels of more than 2-fold within 6 or 18 h, and *3)* a rise in LDLR protein above 3.5-fold within 6 or 18 h. The time column refers to if the effect on LDLR protein levels came early, after 6 h, or only late, after 18 h treatment.

**Fig. 8:**
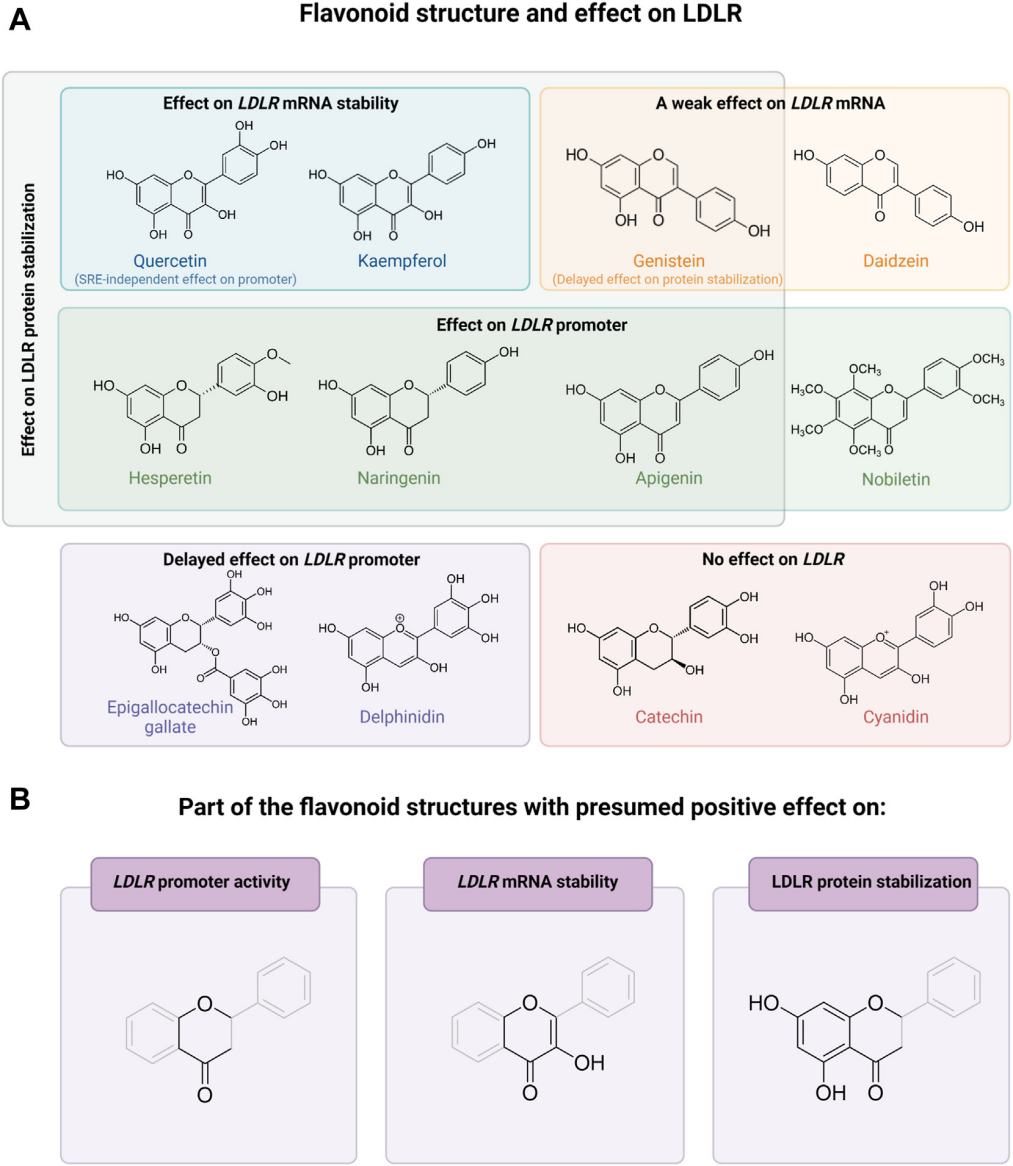
Flavonoid structures and their effects on LDLR. (A) Flavonoids are divided into six groups based on their effects; flavonoids with effect on *LDLR* mRNA stability, with a weak effect on *LDLR* mRNA, with effect on *LDLR* promoter, with delayed effect in *LDLR* promoter, with no effect on *LDLR*, and with effect on LDLR protein stability. (B) Three parts of the flavonoid structures stand out as important for the different effects.

Figures 7 and 8 were made by the use of BioRender.com.

## Results

### The 12 included flavonoids and the basic structure of subclasses

In Fig. 1, we depict all six subclasses of flavonoids along with their basic structure. Two flavonoids from each subclass are included, 12 in total. The concentrations of these flavonoids used in the subsequent experiments were optimized, as explained in the materials and methods section and as shown in Supplemental Figs. S1–S6.

### Effect on LDLR protein levels

The effects of each flavonoid on LDLR protein levels after 6 and 18 h are shown in supplemental Figs. S1 and S4, and the results are depicted in Fig. 2. Although flavonoids have a half-life of 6 h or less in humans, the intermediate products produced by the cells as a response to flavonoid treatment can have a longer half-life. Consequently, 18 h was selected as a suitable timeframe to evaluate the effect on LDLR. All seven flavonoids previously reported to increase LDLR levels did indeed increase LDLR levels after overnight treatment. Six of these (hesperetin, naringenin, kaempferol, quercetin, nobiletin, and epigallocatechin gallate) boosted LDLR levels more than 2-fold. Genistein, in contrast, only exhibited a 64% increase in LDLR protein level after overnight treatment. Noteworthy, a concentration of 50 μM was the only concentration showing a significant increase in LDLR protein level (supplemental Figs. S1 and S4). Among the five flavonoids not previously tested for their effect on LDLR protein levels (apigenin, catechin, cyanidin, delphinidin, and daidzein), only apigenin and delphinidin impacted LDLR protein levels. Both apigenin and delphinidin increased LDLR protein levels more than 2-fold after overnight treatment, with increases of 2.1- and 3.8-fold, respectively.

The results presented in Fig. 2 are derived from a dose–response analysis conducted at 6 and 18 h, as detailed in supplemental Fig. S1 and S4. It is important to note that these results are normalized against their respective controls. Consequently, the quantitative data from these experiments are specific to their conditions and are not directly comparable across different experimental setups. This is due to the relative nature of the measurements, which are specific to the control conditions of each individual assay.

### Flavonoids effect on LDLR mRNA levels

To discern whether the different flavonoids' influence on the LDLR protein level pertains to protein stabilization or a rise in the amount *LDLR* mRNA through enhanced transcription or mRNA stabilization, we assessed their effects on *LDLR* mRNA levels after 6 h and overnight treatments (Fig. 3). All flavonoids that increased LDLR protein levels also increased *LDLR* mRNA levels. As anticipated, cyanidin and catechin had no impact on *LDLR* mRNA levels. However, the two isoflavonoids daidzein and genistein enhanced *LDLR* mRNA levels despite exhibiting little or no effect on LDLR protein levels.

### Effect on the LDLR promoter

Delving deeper into how flavonoids elevate *LDLR* mRNA levels, we employed a luciferase reporter construct featuring the *LDLR* promoter (−1563 bp) and a *LDLR* promoter with a mutated *SRE* sequence. HepG2 cells were cotransfected with a Renilla luciferase plasmid, treated with the various flavonoids, and analyzed for luciferase activity. The results are depicted in Fig. 4. The levels of LDLR promoter activity reflect the effects on *LDLR* mRNA transcription rates. Daidzein and genistein showed a modest effect, aligning with the observed modest increase in *LDLR* mRNA levels (35% and 30%, respectively). Nobiletin, quercetin, delphinidin, and epigallocatechin gallate displayed pronounced effects (between 2- and 5-fold increase in promoter activity), whereas naringenin, apigenin, and hesperetin exhibited a modest but notable increase (between 45% and 83%). Notably, kaempferol did not influence the *LDLR* promoter activity.

All flavonoids that increased *LDLR* promoter activity lost their effect when the *SRE* sequence was mutated, with the exception of quercetin. Interestingly, all flavonoids that increased *LDLR* promoter activity also similarly increased *HMGCR* mRNA levels (supplemental Fig. S7), again with the exception of quercetin. This suggests that hesperetin, naringenin, apigenin, nobiletin, epigallocatechin gallate, delphinidin, daidzein, and genistein all increase *LDLR* promoter activity through its transcription factor SREBP2, known to act on both *HMGCR* and *LDLR*, while quercetin increases *LDLR* promoter activity through *SRE*-independent mechanisms.

### LDLR mRNA stabilization

To determine whether certain flavonoids influenced the amount of *LDLR* mRNA through mRNA stabilization, cells were treated overnight with the different flavonoids, followed by the addition of the transcription inhibitor actinomycin D. Cells were harvested at intervals of 0, 2, 4, and 6 h, RNA was isolated, the levels of *LDLR* mRNA were measured by qPCR analyses (supplemental Fig S8), and the results presented in Fig. 5. Most flavonoids exhibited minimal impact on *LDLR* mRNA stabilization, except the flavanols kaempferol and quercetin. The half-life of *LDLR* mRNA in vehicle-treated cells was 1 h and 31 min, whereas kaempferol increased the *LDLR* mRNA half-life to 2 h and 9 min (42% increase), and quercetin extended it to 3 h and 44 min (an almost 2.5-fold increase). Hesperetin was the only other flavonoid with one time point showing a significant difference compared to the vehicle-treated control. However, hesperetin’s effect on *LDLR* mRNA half-life was a mere 2 min increase compared to the vehicle-treated control.

### LDLR protein stabilization

To investigate whether flavonoids affect LDLR levels through protein stabilization, cells were treated with various flavonoids for 4 hours before adding the protein synthesis inhibitor cycloheximide. The cells were harvested at 0-, 3-, and 6-h intervals, and the levels of LDLR and β-actin were assessed via Western blot analysis (supplemental Figs. S9 and S10), with the results presented in Fig. 6. Six flavonoids—hesperetin, naringenin, kaempferol, quercetin, apigenin, and genistein—positively influenced LDLR protein stability. The other flavonoids showed no impact on LDLR stabilization, except for delphinidin, which had a destabilizing effect. The half-life of LDLR in vehicle-treated cells was 4 h and 9 min. Kaempferol extended the LDLR half-life to 5 h and 17 min (a 27% increase), genistein to 6 h and 30 min (a 32% increase), quercetin to 6 h and 59 min (a 44% increase), hesperetin to 7 h and 59 min (a 92% increase), naringenin to 8 h and 33 min (a 2-fold increase), and apigenin extended LDLR protein stabilization from 4 h and 9 min to 10 h and 51 min (more than a 2.7-fold increase).

### Structure-specific effects on LDLR levels

Figure 7 summarizes our findings, presenting a table that outlines the impacts of various flavonoids on LDLR, detailing their mechanisms of action and the timing of these effects. Notably, within the flavanones subclass, hesperetin and naringenin exhibited nearly identical effects on LDLR levels. The flavonols—kaempferol and quercetin—had similar effects on protein and mRNA stability. However, quercetin had an *SRE*-independent effect on the *LDLR* promoter, whereas kaempferol had no effect on the promoter activity. Similarly, within each subgroup, the flavones (apigenin and nobiletin) and isoflavones (daidzein and genistein) demonstrated consistent effects among their respective flavonoids. However, in both categories, one flavonoid contributed to LDLR protein stabilization, while the other did not. In the flavanol (catechin and epigallocatechin gallate) and anthocyanidin (cyanidin and delphinidin) subclasses, each had one flavonoid affecting LDLR through promoter activity and one that did not. Thus, the effects of the different flavonoids cannot be explained solely by grouping them into subclasses. The question remains: Which parts of the flavonoid structure are responsible for the three distinct effects on LDLR that flavonoids can achieve?

To address this question, we grouped the flavonoid structures based on their effect on LDLR and the timing of this effects (Fig. 8). Genistein, epigallocatechin gallate, and delphinidin exhibited delayed effects, with genistein affecting protein stability later then other flavonoids, and the latter two had a delayed impact on promoter activity (Figs. 4 and 5). These delayed effects likely depend on the cellular metabolic processing and structural changes of the flavonoids, which led us to exclude these structures from comparisons against structures with similar effects.

Three aspects of the flavonoid structures emerged as presumably important for their effects on LDLR levels. Comparing apigenin and kaempferol gave insight into the structural relationship between flavonoids and their effects on LDLR, both of which affects protein stabilization; however, we found that apigenin also impacts *LDLR* promoter activity, while kaempferol affects *LDLR* mRNA stabilization. The distinct structural feature between the two is an OH group at position C3. Quercetin, which possesses this same OH group as kaempferol, also affects *LDLR* mRNA stabilization. In contrast, three other flavonoids with an early and potent effect on *LDLR* promoter activity—hesperetin, naringenin, and nobiletin—have a carbonyl group at position C4, like apigenin, but with no substituents on C3.

Examining structures with effects on LDLR protein stabilization, both ring A and ring C stood out as being important. All flavonoids with OH C5 and C7, in addition to having a carbonyl group at C4, had a positive effect on protein stabilization. The carbonyl group appears crucial since epigallocatechin gallate, delphinidin, catechin, and cyanidin had no effect on protein stabilization. The OH groups also seem important, as nobiletin and daidzein showed no effect.

## Discussion

### Flavonoids and their effect on LDLR

The pivotal role of LDLR in the regulation of plasma cholesterol levels has driven a significant interest in identifying compounds that can modulate its expression. In the present study, various flavonoids were assessed for their potential to influence LDLR protein levels in HepG2 cells. Our findings verify previously published data that hesperetin, naringenin, kaempferol, quercetin, nobiletin, epigallocatechin gallate, and genistein increase LDLR protein levels (21, 22, 23, 24, 25, 26, 27). This increase in LDLR protein level by many of the flavonoids is comparable to, or exceeds, that observed in cells treated with statins (31, 32). Among the flavonoids not previously tested for their effects on LDLR protein levels, apigenin and delphinidin emerged as potent modulators, with the latter showing a remarkable 4-fold rise in LDLR protein level after overnight treatment. Even though daidzein did not have any effect on LDLR protein levels, it did increase *LDLR* mRNA level by 41% after overnight treatment, demonstrating similarities with the other isoflavone, genistein, although being slightly less potent due to its lack of effect on protein stability.

The time-course of the flavonoids' impact on LDLR protein levels was noteworthy. Whereas eight flavonoids demonstrated effect following an overnight treatment, only six influenced LDLR protein level after a 6 h treatment. This discrepancy, evident in the cases of delphinidin and epigallocatechin gallate, hints at the possibility that these flavonoids need metabolic processing outside or within cells before they have an effect on LDLR through increased transcription or that their effects depend on synthesis of new proteins. However, given that the structures of these two flavonoids are similar to those of others that have an early effect on LDLR, it seems more likely that metabolic processing is the underlying factor.

### How structure plays a role in the effect of flavonoids on LDLR

Most of the flavonoids that increased *LDLR* mRNA levels did so through increased promoter activity and heightened *LDLR* mRNA transcription with or without increased LDLR protein stabilization. However, the two flavonoids in the flavonol subclass, kaempferol and quercetin, increased the *LDLR* mRNA levels through increased stability of *LDLR* mRNA. The flavonol structure with a double bond between position 2 and 3, an -OH group attached in position 3, and carbonyl group attached to C4 seems to be important for stabilization of *LDLR* mRNA. Quercetin was more active compared to kaempferol partly due to its *SRE*-independent effect on *LDLR* promoter activity. Thus, a catechol pattern with neighboring OH groups in the B-ring seems to be favorable for this effect.

An early increase of *LDLR* promoter activity as observed for naringenin, hesperetin, nobiletin, and apigenin seems to be dependent on the closed C-ring with carbonyl group in position 4, as well as an aromatic ring attached to C2. Interestingly, apigenin and kaempferol with identical A and B rings showed two different effects on the *LDLR levels*, and the only structural difference between these two compounds is the OH group attached to C3.

Another noticeable difference was observed for epigallocatechin gallate and delphinidin. These substances, representing the flavanol and anthocyanidin subclasses, respectively, showed a delayed effect on the *LDLR* promoter activity. They have in common the pyrogallol groups, aromatic rings with three OH groups attached in meta and para positions. Since a late response was observed, we could speculate if a metabolic process with structural changes of the pyrogallol groups is necessary to obtain an effect. In contrast, catechin and cyanidin, representing the same subclasses as epigallocatechin gallate and delphinidin but without the pyrogallol group, did not show similar effect on *LDLR*, indicating the importance of the pyrogallol group for these subclasses. Also, it indicates that a carbonyl group in C4 is important for an early response.

The isoflavones (genistein and daidzein) showed only modest effects which indicate that the attachment of the B-ring to C3 is unfavorable. Based on a limited number of flavonoids from six different subclasses, there seems to be a few structural elements that are important for *LDLR* activities. We have observed interesting features for the flavonols, especially quercetin, which seems to have an effect on an *SRE*-independent promoter activity, mRNA and protein stability, high activity for methylated flavones at low concentrations, and a delayed response for flavonoids with pyrogallol structural elements. Future studies should take these observations into consideration and explore structural relationships and mechanisms of action of related flavonoids.

### Flavonoids effect on cellular stress

While flavonoids notably impact LDLR protein levels, the exact mechanism influencing *LDLR* expression is unclear. Cellular stress mechanisms, including apoptosis, endoplasmic reticulum-stress, and autophagy, have been associated with elevated *LDLR* mRNA levels due to increased transcription via SREBP2 (33, 34, 35). This raises the possibility that high concentrations of compounds used in treatment may induce cellular stress, which could be the underlying mechanism for the increased transcription of the *LDLR*.

To explore this, our study investigated cell proliferation and viability by using an MTT assay and analyzing apoptosis via PARP cleavage (36, 37). Interestingly, while most flavonoids did alter cell proliferation to some extent, no consistent pattern emerged linking the degree of proliferation to the effect on the LDLR protein level (supplemental Fig. S3). For instance, kaempferol, despite only stabilizing *LDLR* mRNA without affecting its transcription, significantly impeded proliferation by nearly 40% compared to vehicle-treated cells. Nobiletin had the best effect on LDLR protein levels at low concentrations (5 μM) with a mild boost in proliferation, whereas higher concentrations of daidzein (100 μM) did not influence LDLR protein levels but did decrease cell proliferation by approximately 45%.

Our findings indicate that the impact of flavonoids on cell proliferation is not a contributing factor to the increase in LDLR protein levels. Additionally, the results suggest that apoptosis is not a significant factor in this context (supplemental Figs. S2 and S5). Although quercetin, apigenin, and potentially naringenin modestly affected PARP cleavage after overnight treatments, hinting at a possible connection between apoptosis and LDLR upregulation, experiments conducted over shorter durations (6 h) refuted this link. These flavonoids influenced LDLR not solely after overnight treatments but also at the 6 h mark, suggesting that while apoptosis might be involved under specific conditions, it is not the predominant mechanism driving the effects of flavonoids on LDLR protein levels.

### Compounds with similar effects and potentially same target proteins

We have previously demonstrated that two pharmacologic inhibitors of the protein kinase AKT, MK-2206 and triciribine, increase *LDLR* mRNA levels by two different mechanisms (32, 38, 39). MK-2206 stimulates *LDLR* expression by inducing the proteolytic activation of SREBP2, while triciribine enhances *LDLR* mRNA stability. This discrepancy between the two inhibitors, which target the same kinase but by different mode of action, was further confirmed after testing other AKT inhibitors (39). The observation that both different AKT inhibitors and different flavonoids regulate LDLR levels by different mechanisms suggests that slight changes in structure or the mechanism of action are critical for their cellular impact on LDLR. This raises the question: Do compounds that have the same effect on *LDLR* levels operate through the same mechanism or is the LDLR level influenced by a variety of mechanisms and signaling pathways? Future studies should investigate whether flavonoids and AKT inhibitors target the same signaling pathways.

### Flavonoids effect on lipid profiles in vivo

The association between increased flavonoid intake and reduced cardiovascular disease risk has long been recognized, and there is now a growing body of research underscoring flavonoids' role in lipid profile modulation in both humans and animal models (40, 41, 42). Meta-analyses have affirmed that flavonoid treatments or flavonoid-rich diets improved lipid profiles by decreased LDL-cholesterol levels and elevated HDL-cholesterol levels (40, 41, 42). However, the observed reduction in LDL-cholesterol levels is moderate, typically ranging from 0.19 to 0.23 mmol/l (43). This modest impact could partly stem from the methodologies employed in these studies. Human studies often investigate flavonoids within the context of a flavonoid-rich diet, as opposed to administering one pure flavonoid compounds, an approach more common in animal models. While food-based studies reflect real-world flavonoid consumption, they do not precisely study the individual flavonoid effect. The efficacy of flavonoids also hinges on factors such as dosage, overall diet, bioavailability, and large interindividual differences among study participants that are often observed. Additionally, our findings indicate that not all flavonoids are equally potent in regulating LDLR and thus LDL uptake (supplemental Fig. S6), with efficacy varying significantly even among closely related flavonoid subclasses.

The observation of flavonoids' effect on LDL-cholesterol levels in in vivo studies highlights the necessity to deepen our knowledge and identify and optimize the most biologically active and efficient flavonoids. Comprehending the structure-activity relationship is crucial in developing a more potent flavonoid-based treatment for human populations, aimed at enhancing lipid-lowering capabilities and aiding in cardiovascular disease prevention and management. Beyond their lipid-lowering potential, many studies suggest that flavonoids confer cardiovascular protection through additional mechanisms, including anti-inflammatory and antioxidant activities (44). These multifaceted effects should also be considered when optimizing flavonoid-based treatments against cardiovascular diseases, ensuring a holistic approach to harnessing their full therapeutic potential.

### Limitations of our study

Our study has several limitations that merit attention, primarily, our choice of cells. HepG2 cells were chosen to investigate the effect of flavonoids on LDLR due to their human liver origin and inherent expression of LDLR, which is crucial for studying cholesterol metabolism. Their ease of manipulation and the relevance to human hepatic function make HepG2 cells a practical model for initial screenings. While advantageous for preliminary screenings, HepG2 may not fully capture the complexity of in vivo liver physiology due to cell line specificity. This raises concerns about the generalizability of our results, as in vitro conditions lack systemic factors present in an entire organism, such as immune responses and hormonal influences. Additionally, the response to flavonoids in HepG2 cells might differ from other cell lines, like primary hepatocytes, highlighting the need for broader testing to validate our findings.

Our research sheds light on the structural relationship between certain flavonoids and their influence on LDLR. However, it is important to note that flavonoids undergo extensive metabolism following ingestion and absorption in the intestine, involving processes like methylation, glucuronidation, and sulfation (45). There is significant variability in the metabolism of different flavonoids. For example, anthocyanidins exist in plants as glycosides (anthocyanins), and after absorption, these glycosides are metabolized into various glycoside metabolites. Other types of flavonoid glycosides are commonly metabolized into their respective aglycones in the intestine, which are subsequently metabolized in the epithelial and liver cells. Tea catechins (flavanols) are present in plants as aglycones, but also they are extensively conjugated after absorption (46). Due to this extensive metabolism, it is unlikely that the concentration of most flavonoid aglycones used in this study will reach micromolar levels after oral intake. Despite this limitation, our study has identified structural elements, such as the pyrogallol groups in some flavonoids, which appear to enhance the effects on the LDLR levels. Pyrogallol is among the catabolites produced from flavonoids by the colonic microbiota (47), and further investigation is warranted to explore if these flavonoid metabolites can modulate the LDLR levels.

We observed notable variations in the optimal concentrations of the different flavonoids, despite their close structural similarities. Most flavonoids demonstrated the best effect within the range of 50–100 μM. However, distinct variations were evident in two subgroups: flavanones required a higher concentration of 150 μM, while flavones were effective at much lower concentrations of 5–10 μM. Such high concentrations are more comparable to drug-like doses than concentrations that can be achieved by dietary intake alone. However, it is worth noting that a concentration of 5 μM for nobiletin, as used in this study, may be achievable through dietary intake. Nobiletin is classified as a polymethoxylated flavone, meaning that the hydroxyl groups are already methylated. Polymethoxylated flavones have demonstrated to reach μM concentrations in plasma after oral intake (48). The methoxyl groups protect against the conjugation of free hydroxyl groups, resulting in improved metabolic stability and enhanced bioavailability.

Although the concentrations used in this study are hard to achieve through dietary intake, several flavonoids still influenced LDLR levels at lower concentrations (supplemental Fig. S1). The possibility of a synergistic effect from a combination of different flavonoids acting together at these lower concentrations is a compelling avenue for future research.

The differences in optimal concentrations seen after treatment by the 12 flavonoids can be attributed to several factors intrinsic to the molecular nature and interaction dynamics of these compounds. For instance, the position and number of hydroxyl groups can alter their interaction with proteins or signaling molecules that are important for their effect on LDLR levels. While some might have a direct effect, others could potentially modulate their effect through indirect pathways. Additionally, the cellular uptake and distribution of these compounds may vary, affecting the concentration needed to impact LDLR levels. This is further complicated by the metabolic stability of each flavonoid; some are more prone to rapid metabolism or degradation, making higher concentrations necessary for a noticeable effect. The binding affinity and specificity to the key proteins or molecules also play a significant role. For instance, the higher concentration requirement for flavanones could be due to lower affinity or a less direct interaction with these proteins, whereas flavones, requiring lower concentrations, might interact more effectively or have higher stability within the cellular environment. Finally, variations in solubility and bioavailability of each flavonoid class in the cellular milieu are also critical factors. For example, poor solubility might lead to higher required concentrations for some flavonoids. These findings highlight the complexity of flavonoid interactions with biological systems, emphasizing the need for a nuanced understanding of each compound's unique properties.

## Conclusions

Our study accentuates the potential of flavonoids as modulators of LDLR expression and protein function. Their varied effects on the LDLR can be explained by the structural differences among the flavonoids. Our findings particularly emphasize that the flavonol structure with a double bond between position 2 and 3, an -OH group attached in position 3 and carbonyl group attached to C4, plays a crucial role in the stabilization of *LDLR* mRNA. While, the influence of flavonoids on *LDLR* promoter activity appears to be contingent upon the closed C-ring with carbonyl group in position 4, as well as an aromatic ring attached to C2. This is exemplified by our observations of apigenin and kaempferol, where the sole structural distinction of an OH group at C3 leads to divergent effects on LDLR. These insights underscore the necessity for an in-depth exploration into the mechanistic pathways and structure-function relationships of flavonoids in LDLR regulation.

## Data availability

This article contains supplemental data. All data supporting the findings of this study are available within this article and its supplemental data.

## Supplemental data

This article contains supplemental data.

## Conflict of interest

The authors declare that they have no conflicts of interest with the contents of this article.
