# Supplementary material for: Flavonoids regulate LDLR through different mechanisms tied to their specific structures
Source: J Lipid Res. 2024 Mar 29;65(5):100539. doi: 10.1016/j.jlr.2024.100539 (PMC11058080; doi:10.1016/j.jlr.2024.100539)
Supplement: Supplemental Figures S1–S10 [file mmc1.pdf]

Flavonoids regulate LDLR through different mechanisms tied to their specific structures

Supplementary materials

Katrine Bjune, PhD[1*], Pia Skovholt Halvorsen MSc[1#], Helle Wangensteen PhD[2], Trond P. Leren, MD, PhD[1], Martin Prøven Bogsrud, MD, PhD[1] and Thea Bismo Strøm, PhD[1]

[1]Unit for Cardiac and Cardiovascular Genetics, Department of Medical Genetics, Oslo University Hospital, Oslo, Norway
[2]Section for Pharmaceutical Chemistry, Department of Pharmacy, University of Oslo, Oslo, Norway

[#]Current address: Institute for Experimental Medical Research, Oslo University Hospital, Oslo, Norway

*Address correspondence to: Katrine Bjune
Unit for Cardiac and Cardiovascular Genetics
Department of Medical Genetics
Oslo University Hospital
NO-0424 Oslo
Norway

Graphical abstract

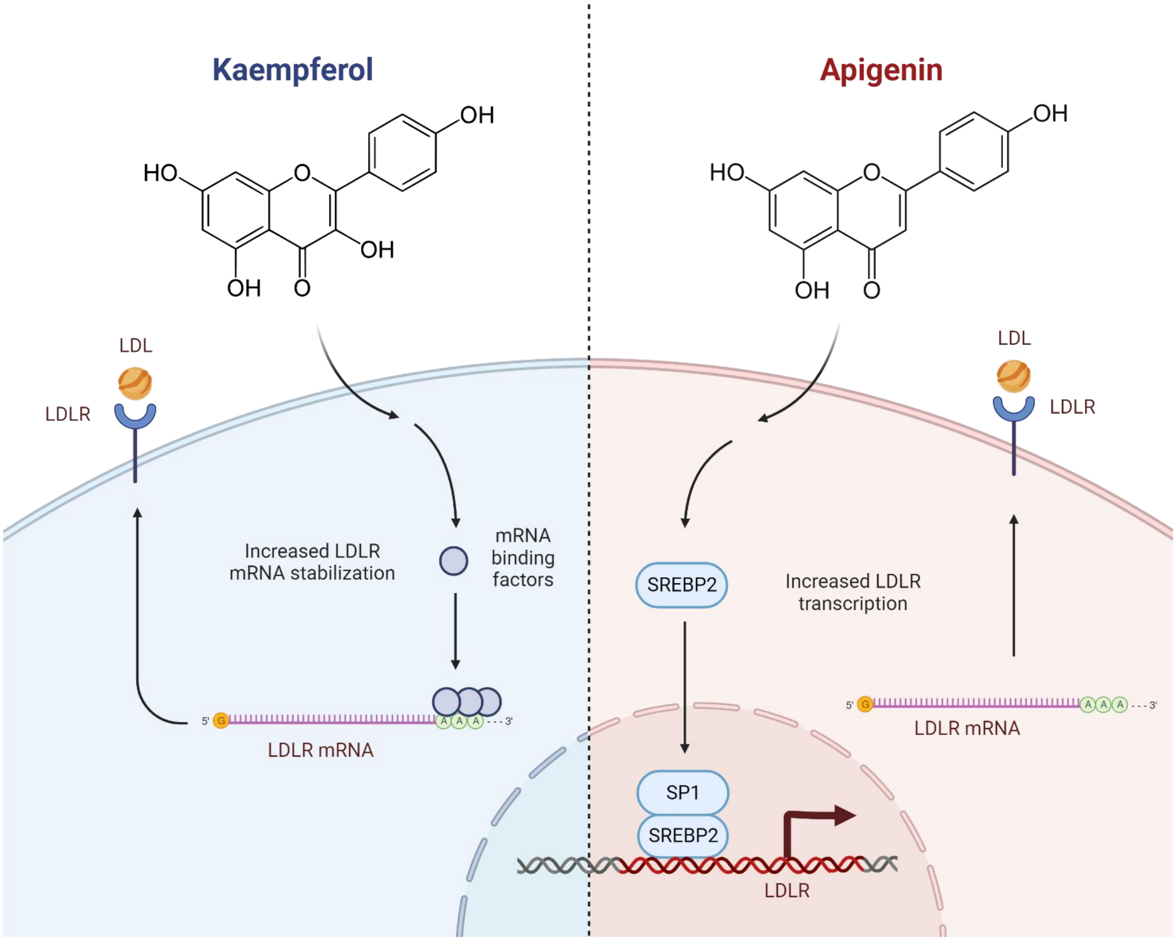

Supplementary figure S1

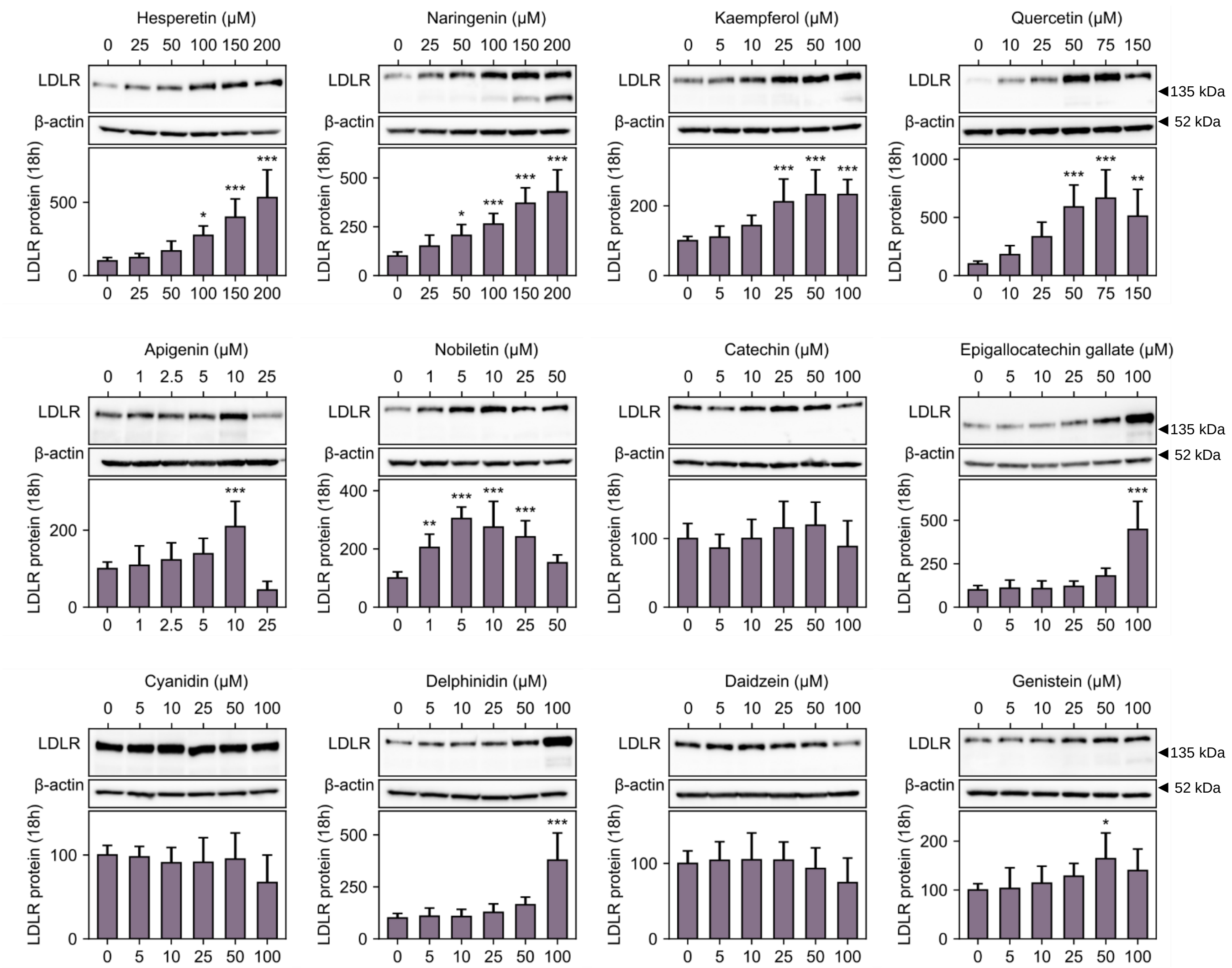

<u>Supplementary figure 1: Dose response of LDLR protein levels after overnight treatment with different flavonoids</u>

HepG2 cells were treated with vehicle or different concentrations of flavonoids overnight (18 hours) before harvesting and determination of LDLR and β-actin protein levels by western blot analyses. LDLR protein levels were corrected for by β-actin levels and plotted relative to the vehicle treated control cells. LDLR levels in vehicle-treated controls (DMSO) (0 μM) and cells incubated with different concentrations of flavonoids are presented as mean +SD from six independent experiments (* $p < 0.05$, ** $p < 0.01$, *** $p < 0.001$, two-tailed t-test vs vehicle treated cells). Each blot is a representative blot from four different experiments.

Supplementary figure S2

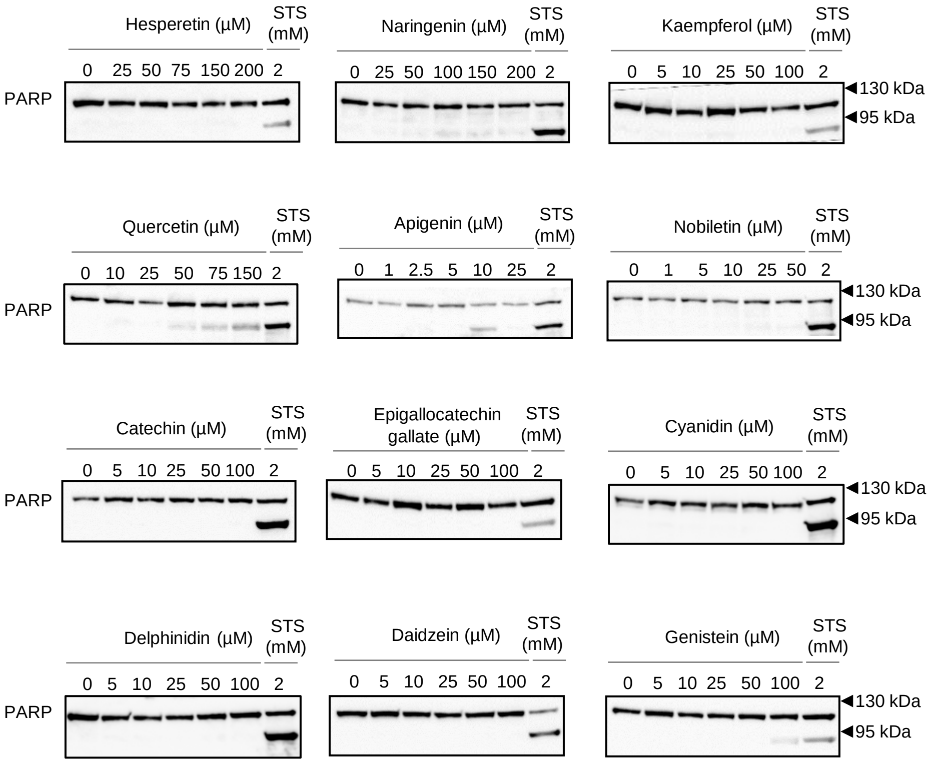

Supplementary figure 2: PARP cleavage as a measurement of apoptosis after 18 h treatment
The same samples as in supplemental figure 1 were also utilized in this figure. HepG2 cells were treated with vehicle or different concentrations of flavonoids overnight before harvesting and determination of Poly (ADP-ribose) polymerase (PARP) levels by western blot analyses. Vehicle-treated controls (DMSO) marked as 0 were used as negative controls, while cells treated with 2 mM staurosporine (STS) for 4 hours were used as a positive control for PARP cleavage. Each blot is a representative blot from four different experiments.

Supplementary figure S3

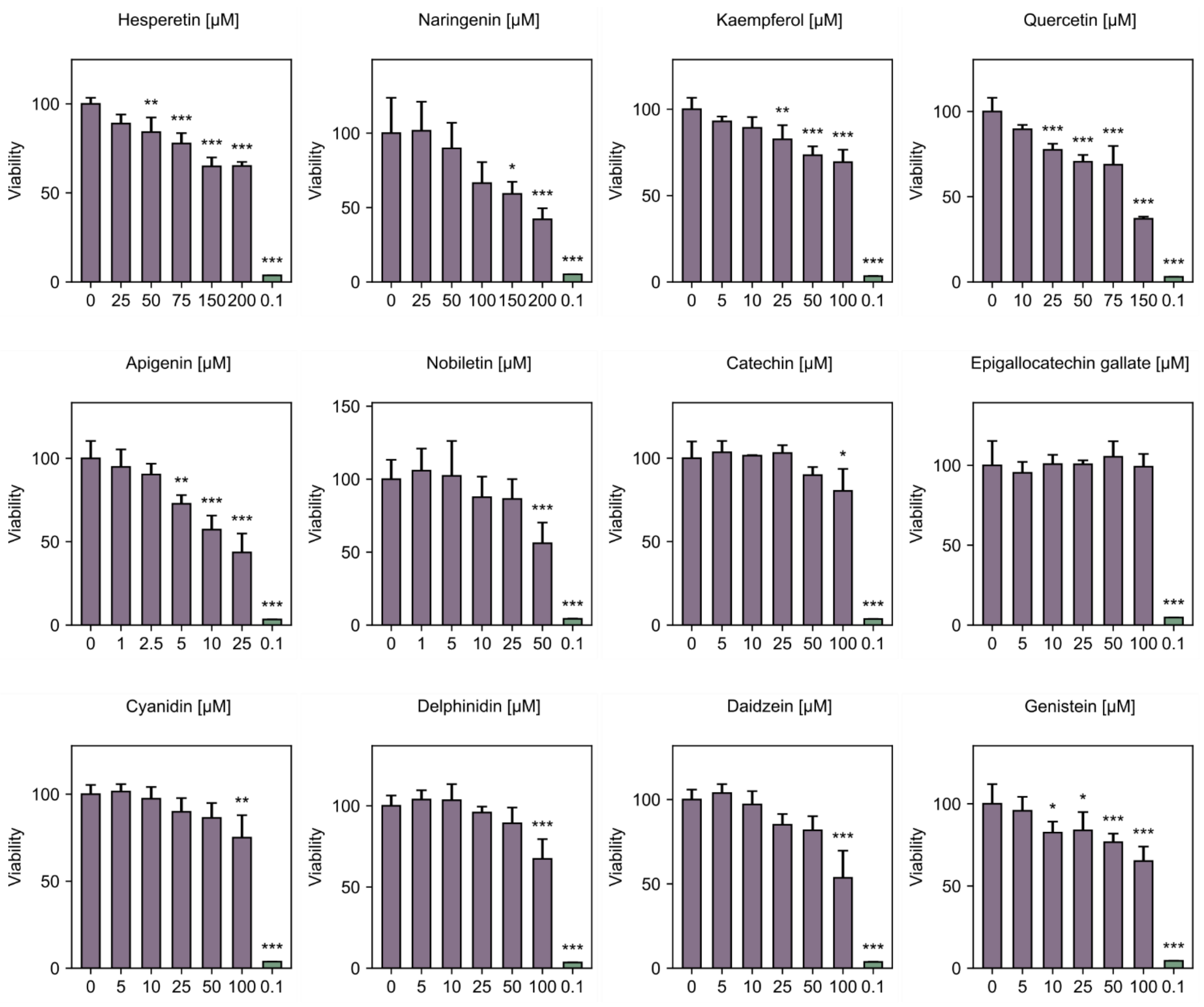

<u>Supplementary figure 3: MTT assay as a measurement of cell viability</u>

HepG2 cells were treated with vehicle (DMSO) (0 µM), different concentrations of flavonoids or 0.1 % triton-100 overnight and the MTT assay was performed according to the manufacturer's instructions. The absorbance in each sample was measured and cell viability was calculated and plotted relative to that of the vehicle treated control cells. The samples are presented as mean +SD from six independent experiments (* $p < 0.05$, ** $p < 0.01$, *** $p < 0.001$, two-tailed t-test vs vehicle treated cells).

Supplementary figure S4

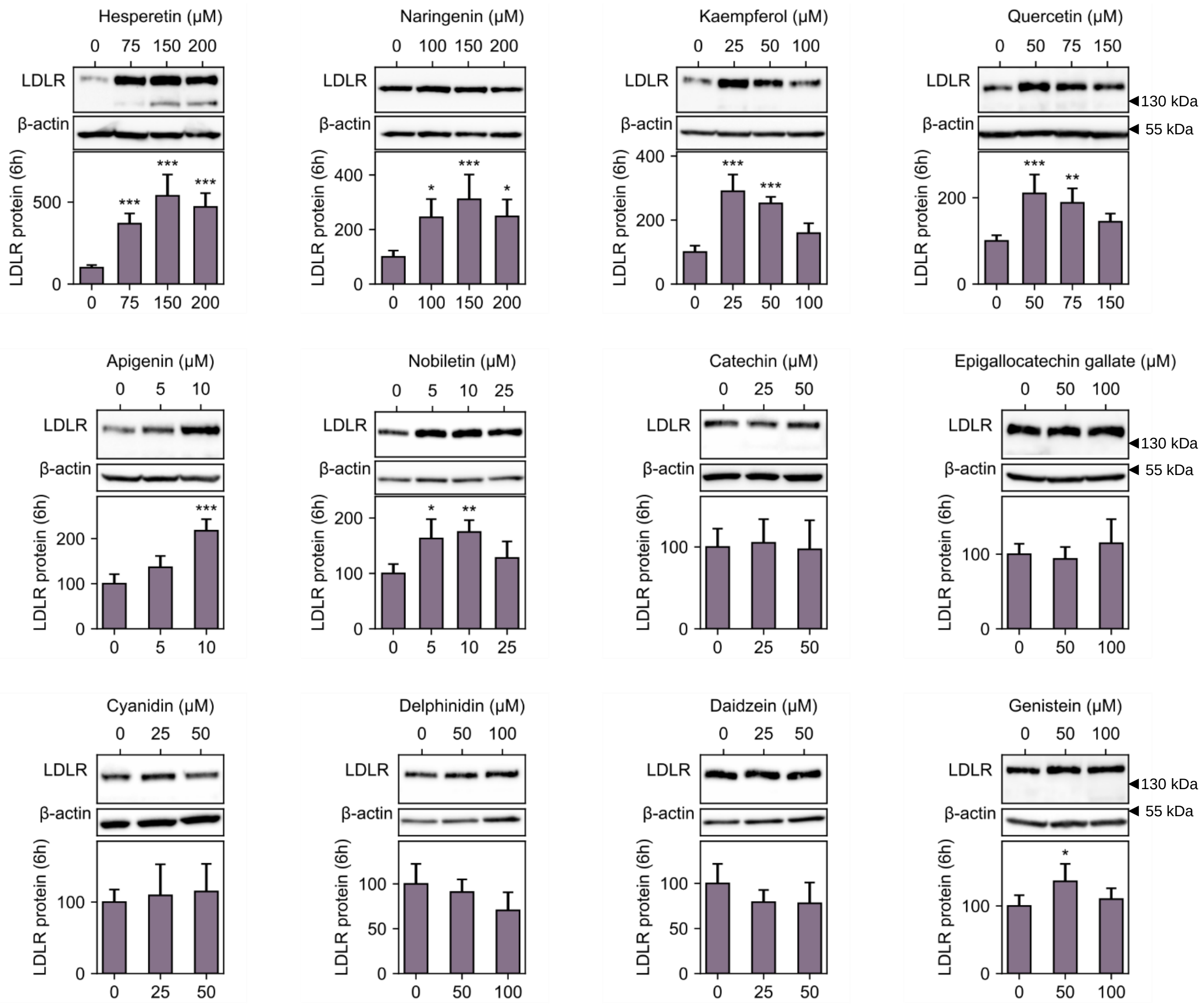

Supplementary figure 4: Dose response of LDLR protein levels after 6 hour treatment with different flavonoids

HepG2 cells were treated with vehicle or different concentrations of flavonoids for 6 hours before harvesting and determination of LDLR and β-actin protein levels by western blot analyses. LDLR protein levels were corrected for by β-actin levels and plotted relative to that of the vehicle-treated control cells. Vehicle treated controls (DMSO) (0 µM) and different concentrations of flavonoids are presented as mean +SD from six independent experiments (* $p < 0.05$, ** $p < 0.01$, *** $p < 0.001$, two-tailed t-test vs vehicle treated cells). Each blot is a representative blot from four different experiments.

Supplementary figure S5

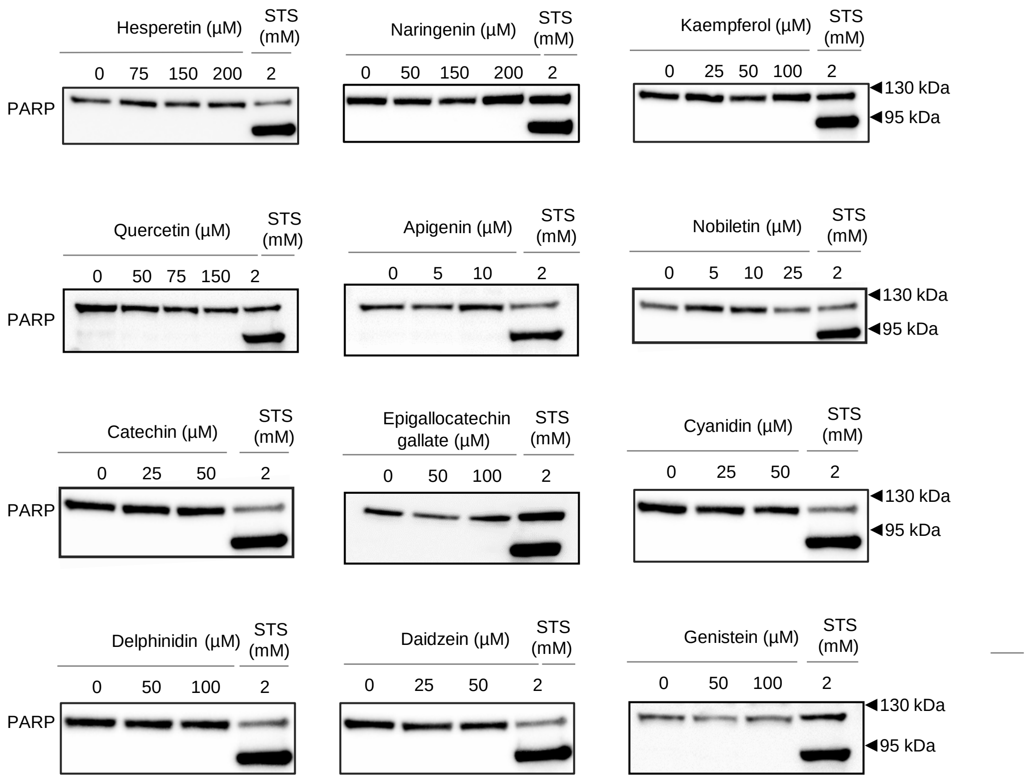

<u>Supplementary figure 5: PARP cleavage as a measurement of apoptosis after 6 hour treatment</u>
The same samples as in supplemental figure 4 were also utilized in this figure. HepG2 cells were treated with vehicle or different concentrations of flavonoids for 6 h before harvesting and determination of Poly (ADP-ribose) polymerase (PARP) levels by western blot analyses. Vehicle treated controls (DMSO) marked as 0 were used as negative controls, while cells treated with 2 mM staurosporine (STS) for 4 hours were used as a positive control of PARP cleavage. Each blot in this figure is shown as one representative blot from four different experiments.

Supplementary figure S6

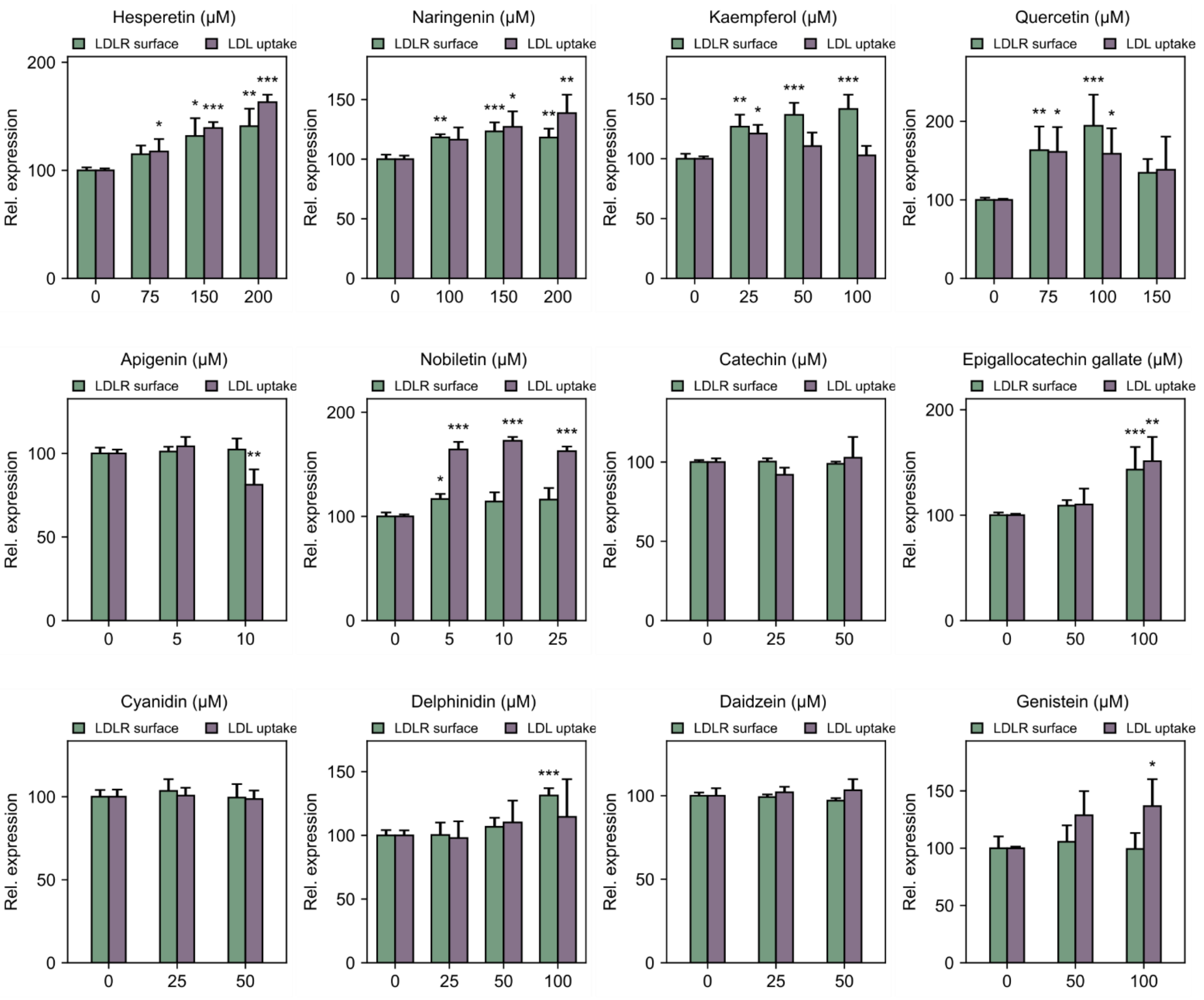

<u>Supplementary figure 6: LDLR on the cell surface and LDL-uptake after flavonoid treatment</u>

This figure contains results from two different experiments, light blue bars represent the relative level of LDLR protein on the cell surface, and the dark blue bars represent did-LDL uptake. In both experiments, HepG2 cells were treated with either vehicle or different flavonoids overnight (18 h).

Supplementary figure S7

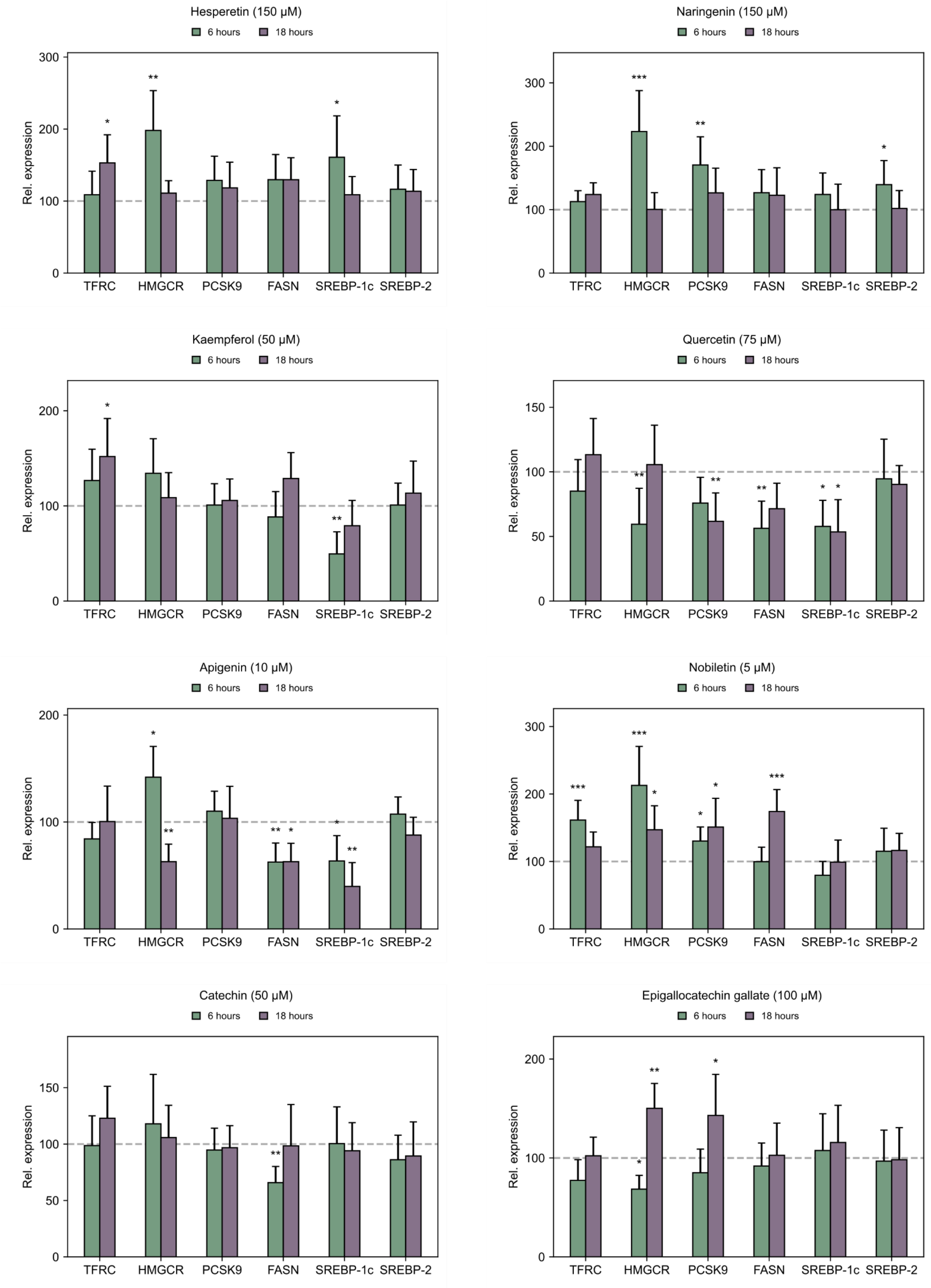

Supplementary figure S7

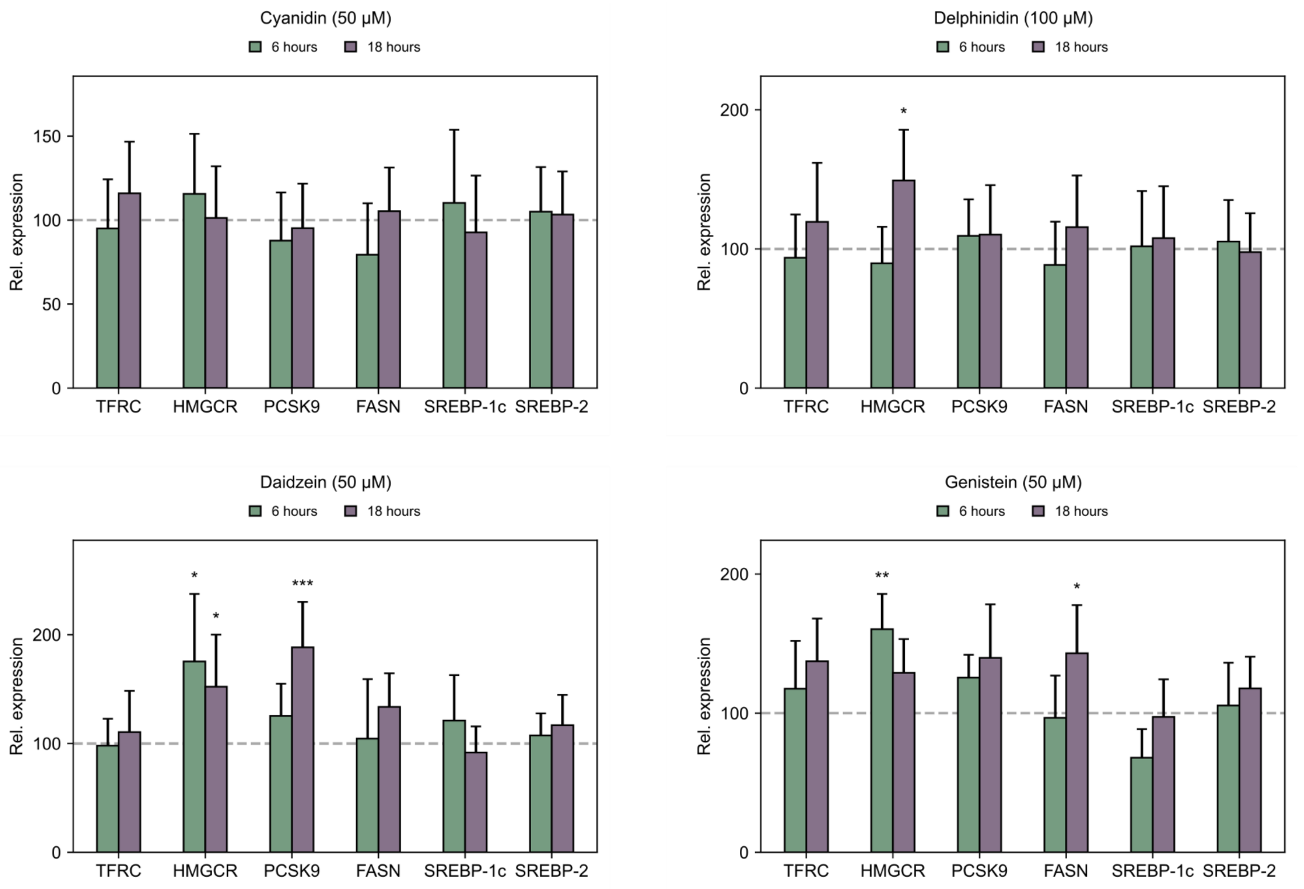

<u>Supplementary figure 7: mRNA levels after 6 hours or 18 hours treatment with different flavonoids</u>

HepG2 cells were treated with vehicle or different flavonoids for 6 hours (light blue) or 18 hours (dark blue) before harvesting and determination of mRNA levels by qPCR. The mRNA levels analysed were transferrin receptor (*TFRC*), 3-hydroxy-3-methylglutaryl-CoA reductase (*HMGCR*), proprotein convertase subtilisin/kexin type 9 (*PCSK9*), fatty acid syntetase (*FASN*), sterol regulatory element binding protein 1c (*SREBP1c*) and sterol regulatory element binding protein 2 (*SREBP2*). The different levels of mRNA were plotted relative to their internal control, vehicle treated control cells (DMSO) which were assigned a value of 100. The vehicle treated control samples are not shown, but are indicated by the gray dashed line. The different mRNA levels are presented as mean +SD from six independent experiments (* $p < 0.05$, ** $p < 0.01$, *** $p < 0.001$).

Supplementary figure S8

Vehicle									
0h	1,000	1,000	1,000	1,000	1,000	1,000	1,000	1,000	1,000
2h	0,375	0,352	0,293	0,325	0,401	0,328	0,323	0,401	0,321
4h	0,190	0,194	0,132	0,184	0,195	0,184	0,198	0,195	0,192
6h	0,105	0,097	0,124	0,096	0,120	0,096	0,127	0,120	0,112
Catechin									
0h	1,000	1,000	1,000	1,000					
2h	0,316	0,361	0,249	0,263					
4h	0,157	0,140	0,094	0,141					
6h	0,070	0,083	0,094	0,079					
Hesperetin									
0h	1,000	1,000	1,000	1,000					
2h	0,409	0,459	0,476	0,476					
4h	0,237	0,202	0,164	0,126					
6h	0,169	0,134	0,161	0,135					
Naringenin									
0h	1,000	1,000	1,000	1,000					
2h	0,486	0,410	0,418	0,525					
4h	0,196	0,178	0,116	0,142					
6h	0,154	0,118	0,108	0,103					
Delphinidin									
0h	1,000	1,000	1,000	1,000	1,000				
2h	0,263	0,293	0,306	0,230	0,214				
4h	0,104	0,085	0,142	0,106					
6h	0,116	0,107	0,098	0,083					
Apigenin									
0h	1,000	1,000	1,000	1,000	1,000	1,000			
2h	0,246	0,536	0,307	0,241	0,328	0,419			
4h	0,111	0,215	0,117	0,115					
6h	0,067	0,122	0,084	0,085					
Kaempferol									
0h	1,000	1,000	1,000	1,000	1,000				
2h	0,428	0,475	0,491	0,578					
4h	0,342	0,338	0,229	0,252	0,282				
6h	0,135	0,220	0,193	0,103	0,127				
Nobelitin									
0h	1,000	1,000	1,000	1,000	1,000				
2h	0,276	0,300	0,304	0,300	0,434				
4h	0,094	0,212	0,116	0,164	0,133				
6h	0,079	0,164	0,094	0,090	0,092				
Daidzein									
0h	1,000	1,000	1,000	1,000	1,000				
2h	0,200	0,403	0,295	0,338	0,440				
4h	0,131	0,165	0,161	0,166	0,250				
6h	0,079	0,117	0,063	0,132	0,131				
Cyanidin									
0h	1,000	1,000	1,000	1,000	1,000				
2h	0,356	0,287	0,409	0,374	0,549				
4h	0,142	0,132	0,189	0,235	0,174				
6h	0,081	0,102	0,158	0,133	0,082				
Epigallocatechin gallate									
0h	1,000	1,000	1,000	1,000	1,000	1,000			
2h	0,319	0,297	0,354	0,357	0,370	0,307			
4h	0,081	0,138	0,158	0,152	0,115				
6h	0,089	0,120	0,125	0,149	0,115				
Genistein									
0h	1,000	1,000	1,000	1,000	1,000	1,000			
2h	0,198	0,434	0,516	0,265	0,509	0,214			
4h	0,168	0,181	0,217	0,159	0,269				
6h	0,083	0,154	0,152	0,118	0,173				
Quercetin									
0h	1,000	1,000	1,000	1,000	1,000	1,000			
2h	0,418	0,683	0,788	0,697	0,468				
4h	0,350	0,334	0,476	0,669	0,458	0,485			
6h	0,435	0,451	0,339	0,425	0,354	0,275			

Supplementary figure 8: : LDLR mRNA stability after treatment with different flavonoids
This is an overview of all data points from all experiments included in figure 5.

Supplementary figure S9

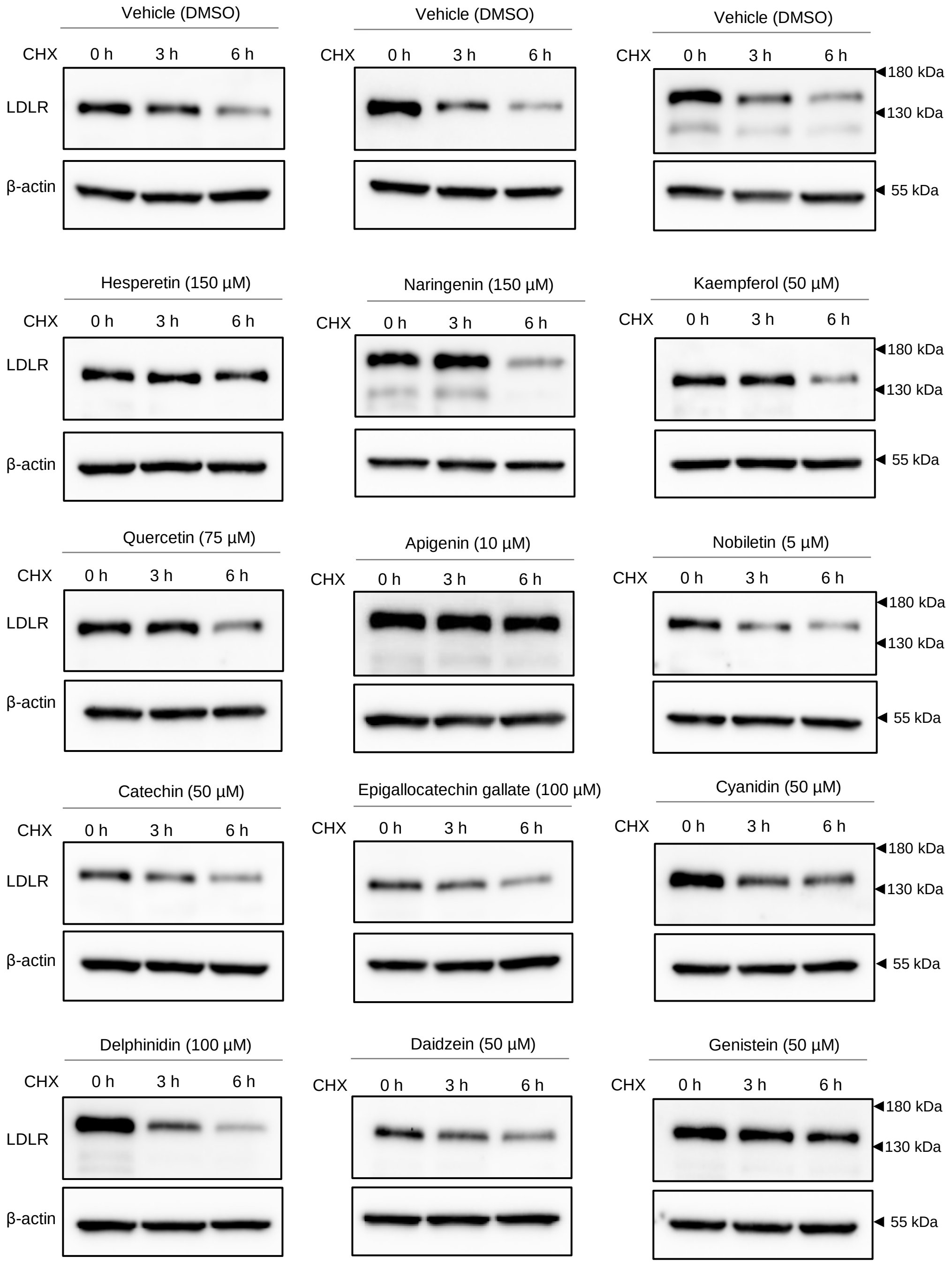

<u>Supplementary figure 9: Flavonoids effect on LDLR protein levels after cycloheximide treatment</u>
HepG2 cells were treated with vehicle or different flavonoids for 4 hours before cycloheximide (CHX) treatment. The cells were harvested after 0, 3 and 6 hours and LDLR levels were determined by western blot. This is one representative blot out of five. The quantification are shown in figure 5.

Supplementary figure S10

		1	2	3	4	5	6	Average	SD	Average	SD
DMSO	0 h	0.61	0.79	0.76	0.60	0.73	0.76	0.71	0.11	100.00	15.60
	3 h	0.35	0.29	0.29	0.49	0.55	0.55	0.42	0.12	59.44	17.86
	6 h	0.35	0.25	0.20	0.10	0.29	0.46	0.28	0.11	39.00	16.28
DMSO	0 h	0.63	0.91	0.61	0.81	0.77	0.87	0.77	0.12	100.00	15.74
	3 h	0.33	0.24	0.48	0.43	0.43	0.63	0.42	0.13	55.00	17.02
	6 h	0.23	0.14	0.13	0.31	0.35	0.55	0.28	0.16	37.13	20.40
DMSO	0 h	0.40	0.52	0.45	0.76	0.74	0.59	0.58	0.14	100.00	24.36
	3 h	0.28	0.47	0.26	0.26	0.34	0.53	0.36	0.12	61.58	20.50
	6 h	0.24	0.48	0.14	0.11	0.24	0.12	0.22	0.06	38.43	10.46
Apigenin	0 h	0.94	0.91	0.76	0.71	0.89		0.84	0.10	100.00	11.92
	3 h	0.82	0.48	0.57	0.77	0.83		0.70	0.16	82.65	18.65
	6 h	0.72	0.47	0.39	0.65	0.65		0.57	0.14	68.16	16.44
Catechin	0 h	0.74	0.78	0.91	0.62	0.58		0.73	0.13	100.00	18.16
	3 h	0.37	0.54	0.69	0.25	0.43		0.46	0.17	63.20	23.16
	6 h	0.33	0.41	0.41	0.20	0.17		0.30	0.11	41.49	15.62
Cyanidin	0 h	0.72	0.90	0.57	0.49	0.77		0.69	0.16	100.00	23.50
	3 h	0.34	0.27	0.55	0.49	0.47		0.42	0.11	61.31	16.59
	6 h	0.24	0.13	0.17	0.15	0.31		0.20	0.07	28.99	10.30
Daidzein	0 h	0.48	0.35	0.32	0.53	0.50		0.44	0.10	100.00	21.85
	3 h	0.29	0.21	0.17	0.35	0.39		0.28	0.09	65.09	21.52
	6 h	0.10	0.13	0.13	0.22	0.29		0.18	0.08	40.44	18.41
Delphinidin	0 h	0.46	0.49	0.54	0.68	0.48		0.53	0.09	100.00	16.79
	3 h	0.33	0.25	0.26	0.21	0.16		0.24	0.06	45.84	12.18
	6 h	0.12	0.08	0.14	0.06	0.03		0.09	0.04	16.21	8.41
Epigallocatechin	0 h	0.49	0.55	0.45	0.67	0.66		0.57	0.10	100.00	17.55
gallate	3 h	0.36	0.25	0.47	0.31	0.45		0.37	0.09	64.97	16.65
	6 h	0.08	0.07	0.11	0.07	0.24		0.11	0.07	20.21	12.68
Genistein	0 h	0.88	0.68	0.73	0.66	0.62		0.71	0.10	100.00	13.95
	3 h	0.36	0.49	0.46	0.45	0.62		0.48	0.09	66.50	13.24
	6 h	0.25	0.43	0.42	0.38	0.50		0.39	0.09	55.11	13.18
Hesperetin	0 h	0.74	0.97	0.71	0.85	0.80		0.81	0.10	100.00	12.60
	3 h	0.66	0.65	0.68	0.76	0.53		0.66	0.08	80.65	9.97
	6 h	0.55	0.30	0.55	0.67	0.29		0.47	0.17	58.23	21.04
Kaempferol	0 h	0.77	0.57	0.80	0.76	0.75		0.73	0.09	100.00	14.70
	3 h	0.68	0.55	0.42	0.66	0.64		0.59	0.11	81.11	17.46
	6 h	0.18	0.35	0.26	0.27	0.37		0.28	0.08	39.02	12.67
Naringenin	0 h	0.34	0.49	0.34	0.36	0.28		0.36	0.08	100.00	21.89
	3 h	0.33	0.43	0.37	0.27	0.32		0.35	0.06	95.45	15.95
	6 h	0.17	0.29	0.09	0.26	0.22		0.20	0.08	56.30	21.86
Nobiletin	0 h	0.12	0.16	0.13	0.14	0.22	0.15	0.15	0.03	100.00	21.53
	3 h	0.08	0.08	0.09	0.07	0.11	0.10	0.09	0.01	58.01	8.60
	6 h	0.06	0.04	0.02	0.05	0.10	0.09	0.06	0.03	39.55	19.00
Quercetin	0 h	0.44	0.39	0.37	0.44	0.36	0.40	0.40	0.03	100.00	8.14
	3 h	0.38	0.32	0.48	0.44	0.27	0.24	0.35	0.10	88.09	23.67
	6 h	0.20	0.14	0.16	0.33	0.16	0.21	0.20	0.07	50.04	17.17

Supplementary figure 10: : Flavonoids effect on LDLR protein levels after cycloheximide treatment
This is an overview of all data points from all experiments included in figure 6.
